# Ubiquitination‐Dependent LLGL2 Degradation Drives Colorectal Cancer Progression via *THBS3* mRNA Stabilization

**DOI:** 10.1002/advs.202501656

**Published:** 2025-07-06

**Authors:** Jiayan Huang, Tiantian Zhang, Huimin Li, Zidan Li, Shuangshuang Yin, Yiman Liu, Chunze Zhang, Yuling Qiu, Haiyang Yu

**Affiliations:** ^1^ State Key Laboratory of Chinese Medicine Modernization Tianjin University of Traditional Chinese Medicine Tianjin 301617 China; ^2^ Haihe Laboratory of Modern Chinese Medicine Tianjin 301617 China; ^3^ Department of Colorectal Surgery, Tianjin Union Medical Center Nankai University Tianjin 300122 China; ^4^ School of Pharmacy Tianjin Medical University Tianjin 300070 China

**Keywords:** colorectal cancer, LLGL2, PI3K‐Akt, THBS3, ubquitination

## Abstract

Colorectal cancer (CRC) is the second most common cause of cancer‐related deaths worldwide is highly associated with distant organ metastasis. Lethal(2) giant larvae protein homolog 2 (LLGL2) is often dysregulated in various tumors; however, the pathogenesis of CRC remains unclean. This study highlighted the tumor suppressor function of LLGL2 in CRC. Depleted LLGL2 exhibits the pro‐CRC effects. RNA sequencing reveals that LLGL2 suppresses CRC progression by inhibiting the phosphoinositide 3‐kinase (PI3K)‐protein kinase B (PKB)/Akt pathway. Further analysis using RNA immunoprecipitation sequencing and shotgun mass spectrometry indicated that LLGL2 primarily regulates the stability of thrombospondin 3 (*THBS3)* mRNA by interacting with CCR4‐NOT transcription complex subunit 1 (CNOT1), thus inactivating the PI3K‐Akt pathway. Additionally, MDM2 acts as an upstream modulator of LLGL2 and promotes its degradation via the proteasomal pathway. This novel mechanism reveals potential therapeutic targets for CRC treatment and enhanced the understanding of how CRC progression can be controlled.

## Graphical abstract

During the progression of CRC, MDM2, as an E3 ubiquitin ligase, promotes the degradation of the LLGL2 protein. Reduced expression of the LLGL2 protein leads to the loss of support for the CNOT1 protein, decreasing the degradation of *THBS3* mRNA. The increased THBS3 further activates the PI3K‐Akt pathway, promoting the proliferation and metastasis of CRC.

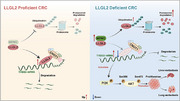

## 1 Introduction

Colorectal cancer (CRC) is the second most common cause of cancer‐related death, accounting for approximately 900000 deaths annually.^[^ ^1^ ^]^ Distant metastasis substantially contributes to mortality associated with CRC, with the liver and lungs being the most frequently affected organs.^[^ ^2^ ^]^ The anatomical connection between the liver and the intestine through the portal vein renders the liver the most common site of CRC metastasis.^[^ ^3^ ^]^ Currently, effective treatment methods for colon cancer, particularly metastatic colon cancer, are lacking. Therefore, it is important to investigate the pathological mechanisms and key molecules involved in the development and progression of colon cancer, as well as develop targeted therapeutic drugs that can prolong patient survival and improve their quality of life.

The larval giant larvae (LGL) family was initially found and identified in *Drosophila* and is a critical component of the scribble cell polarity protein complex. It plays an essential role in maintaining the apical‐basal polarity in epithelial cells and facilitating asymmetric cell division.^[^ ^4^ ^]^ Lethal(2) giant larvae protein homolog 2 (LLGL2) encodes a scaffold protein that regulates cell polarity and contributes to the maintenance of apical‐basal polarity.^[^ ^5^, ^6^, ^7^ ^]^ An imbalance in the distribution of polar proteins can disrupt cell polarity, thereby promoting tumor initiation, proliferation, and migration. However, the relationship between polar proteins and onset and progression of human tumors remains underexplored in current unclear.^[^ ^8^ ^]^ Existing research indicates that LLGL2 expression is dysregulated in various tumor tissues, suggesting its potential role as a tumor suppressor.^[^ ^9^, ^10^, ^11^ ^]^ LLGL2 expression gradually decreases during gastric cancer development.^[^ ^12^ ^]^ In ovarian cancer, LLGL2 expression is reduced and it interacts with Alpha ‐ actinin ‐ 1 (ACTN1), altering its intracellular localization and preventing the aggregation of actin filaments into bundles. This interaction leads to cytoskeletal remodeling, thereby inhibiting the malignant behavior of cancer cells.^[^ ^13^ ^]^ In breast cancer, LLGL2 expression is decreased and restoring LLGL2 expression can inhibit snail‐driven epithelial‐mesenchymal transition (EMT).^[^ ^14^ ^]^ Additionally, LLGL2 is significantly overexpressed in estrogen receptor‐positive (ER^+^) breast cancer, which facilitates cell proliferation under nutritional stress. LLGL2 also interacts with SLC7A5 and YKT6 to mediated the amino acid transport pathway, thereby contributing to the development of resistance to tamoxifen resistance.^[^ ^15^ ^]^ In CRC, LLGL2 deficiency is associated with enhanced EMT and metastasis in CRC, although the underlying mechanism remains elusive.^[^ ^16^ ^]^ These findings demonstrated the complex expression patterns of LLGL2 in cancer cells. LLGL2 may primarily function as a tumor suppressor in CRC, influencing tumorigenesis and progression, although the precise mechanisms remain to be elucidated.

RNA‐binding proteins (RBPs) interacted with downstream target RNAs to form complexes critical for the precise regulation of RNA metabolism related to oncogenes and tumor suppressors.^[^ ^17^, ^18^ ^]^ LLGL2, containing multiple WD40 repeats, acts as a scaffold for RNA interactions—binding the long non‐coding RNA (lncRNA) MAYA via its first three WD40 domains and recruiting SUN6 to promote MST1 methylation, thereby activating the YAP pathway and driving bone metastasis.^[^ ^19^, ^20^, ^21^ ^]^ This suggests that LLGL2 acts as an RBP in tumors. Whether LLGL2 functions as an RBP to regulate downstream RNAs in CRC has not been previously reported and remains unclear.

To further explore the role of LLGL2 in CRC, we performed RNA immunoprecipitation sequencing (RIP‐seq) and RNA sequencing (RNA‐seq). Our findings indicate that LLGL2 interacts with the mRNA of thrombospondin 3 (*THBS3*) and modulates its expression. THBS3 is an extracellular matrix glycoprotein that facilitates cell‐cell interactions.^[^ ^22^ ^]^ The TSP family can facilitate the progression of various cancers, including gastric, colorectal, and prostate cancers.^[^ ^23^, ^24^, ^25^ ^]^ However, there are currently few reports on the role of THBS3 in the context of cancer, and its underlying mechanisms remain unclear. Our results indicate that THBS3 expression is elevated in CRC, which functions as a downstream mediator of LLGL2 by modulating the PI3K‐Akt pathway. To investigate the molecular mechanisms underlying LLGL2 downregulation in CRC, we integrated bioinformatic analyses with experimental validation. Our findings demonstrate that LLGL2 undergoes proteasomal degradation mediated by ubiquitination, with MDM2 being identified as the primary E3 ubiquitin ligase responsible for this post‐translational modification. Therefore, therapeutic strategies targeting MDM2 inhibition or LLGL2 activation represent a promising approaches for the treatment of CRC.

## 2 Results

### 2.1 LLGL2 was Downregulated in CRC and low LLGL2 was Associated with Poor Prognosis

**Figure 1:**
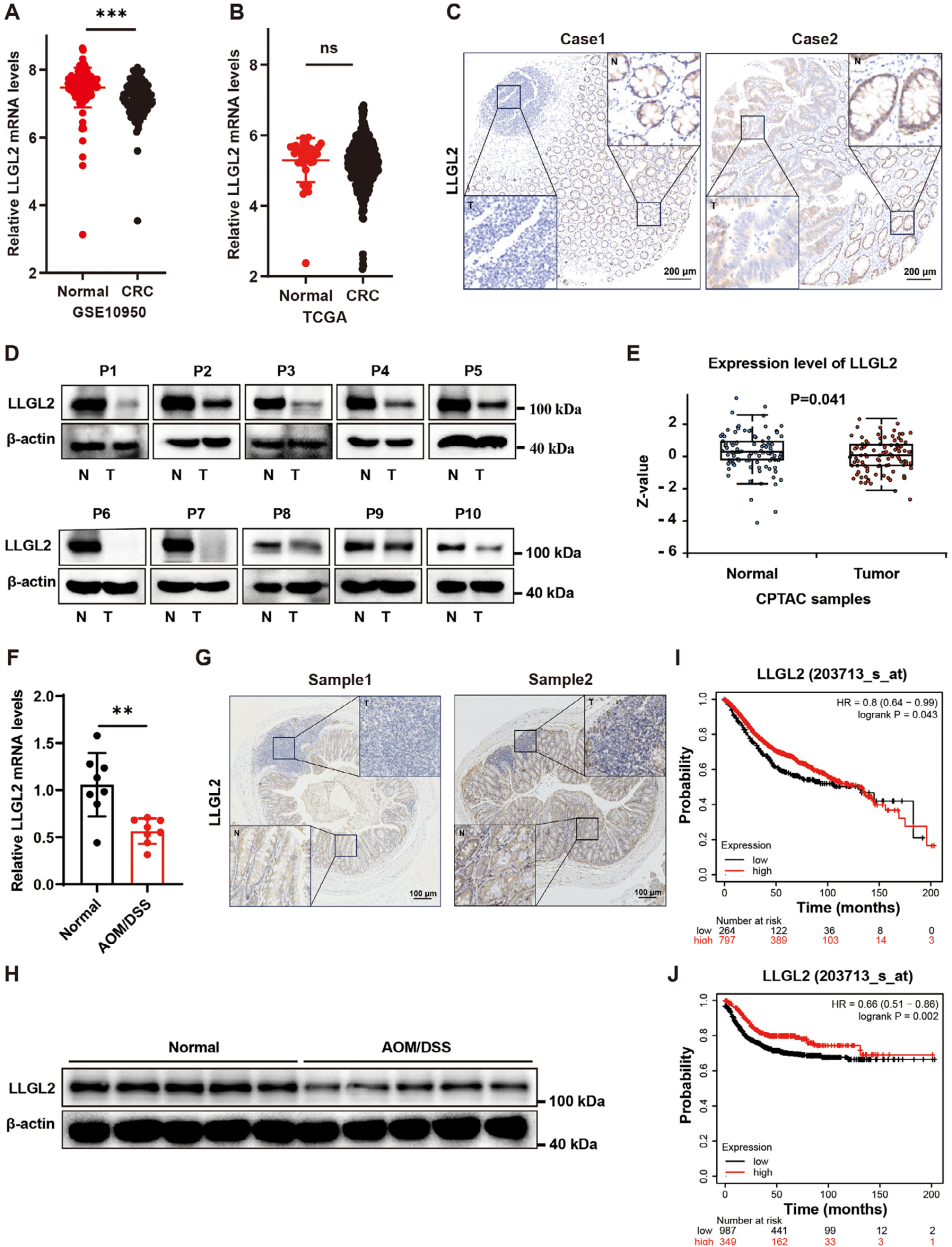
LLGL2 was downregulated in CRC and low LLGL2 was associated with poor prognosis. A) Gene chip data of LLGL2 was obtained from GEO dataset. B) The TCGA dataset indicating the expression levels of LLGL2 within CRC. C) Representative IHC images of LLGL2 expression in CRC and normal tissues. Scale bar, 200 µm. D) LLGL2 expression was analyzed in 10 pairs of CRC tissues from patient. (N for non‐tumor tissue, T for tumor tissue). E) The protein expression data of LLGL2 was from CPTAC. F) RT‐qPCR analysis of LLGL2 expression in colon tissue of mice with and without AOM/DSS treatment. G) Representative LLGL2 IHC staining of colon tissue from AOM/DSS‐treated mice, scale bar, 100 µm. H) Western blotting analysis of LLGL2 expression in colon tissues from AOM/DSS‐induced and control mice. I) Kaplan–Meier analysis of OS in patients with high versus low LLGL2 expression J) Kaplan– Meier analysis of RFS in patients with high versus low LLGL2 expression. Data are presented as mean ± SD. *P*‐values are determined by a two‐tailed Student's *t*‐test. **P < 0.01, ***P < 0.001, ns (not significant).

To elucidate the LLGL2 expression in CRC, we first assessed the expression levels of LLGL2 using public databases. Results from the GEO (GSE10950) database revealed a significant reduction in *LLGL2* mRNA expression in CRC tissues compared with that in normal tissues (**Figure** **1**A). Additionally, TCGA data showed no significant differences in *LLGL2* transcript levels between CRC and non‐tumor tissues (Figure 1B). Immunohistochemistry (IHC) and western blotting analyses of patient CRC tissues further demonstrated a decrease in LLGL2 protein expression (Figure 1C,D). Moreover, the CPTAC data corroborated the downregulation of LLGL2 protein expression in CRC tissues (Figure 1E). In the colon tissues of Azoxymethane/dextran sulfate sodium (AOM/DSS)‐induced CRC mice, decreased expression of LLGL2 was also observed (Figure 1F). IHC and western blotting assays further confirmed the loss of LLGL2 protein expression in these AOM/DSS‐induced CRC mice (Figure 1G,H). Kaplan–Meier survival analysis revealed that reduced LLGL2 expression was positively correlated with shorter overall survival (OS) and relapse‐free survival (RFS) (Figure 1I,J). Collectively, these findings indicate that LLGL2 is downregulated in CRC and diminished LLGL2 expression is correlated with an unfavorable prognosis.

### 2.2 The Knockdown of LLGL2 Enhances The Proliferation and Metastasis of CRC Cells Both In Vivo and In Vitro

**Figure 2:**
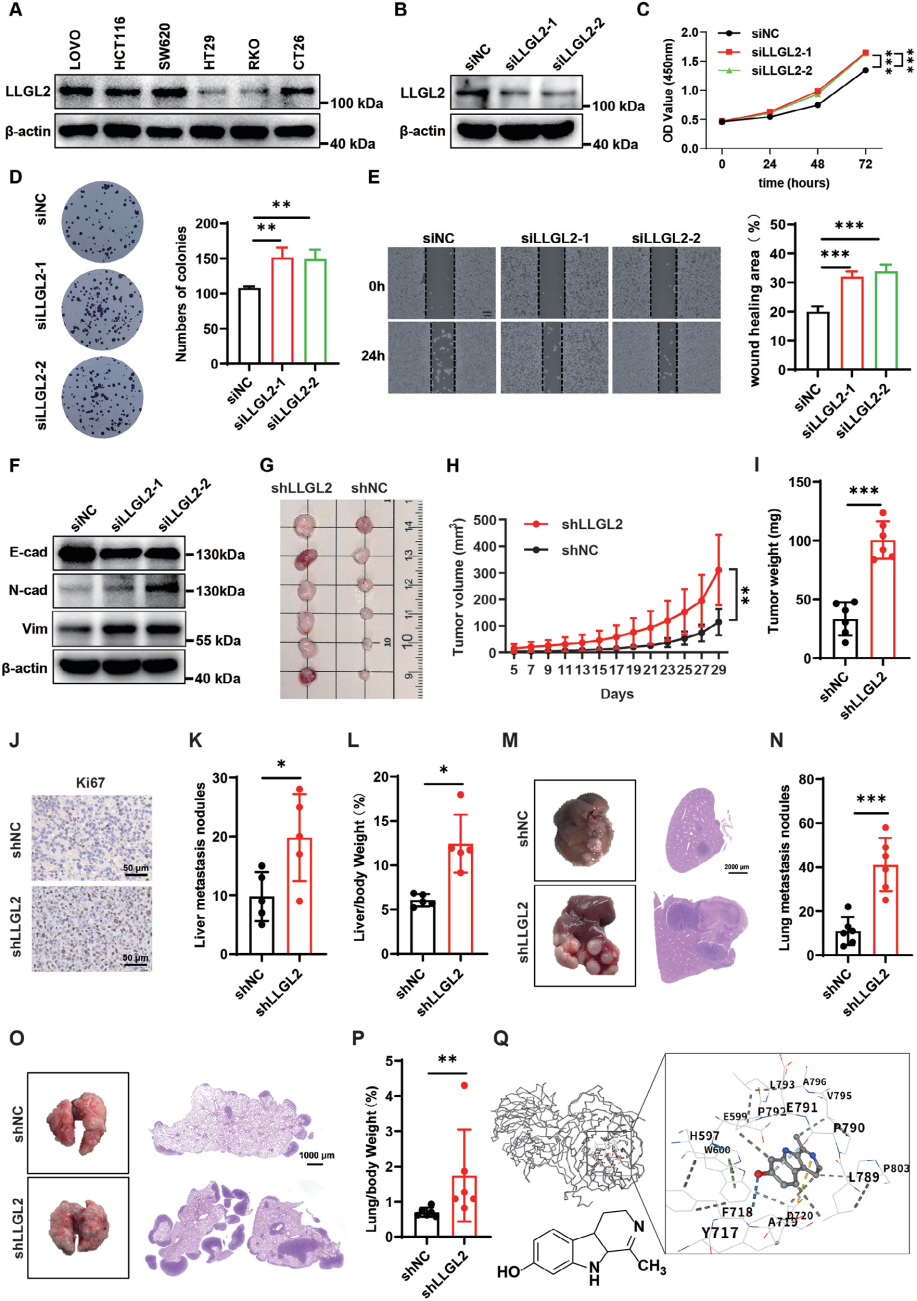
The knockdown of LLGL2 enhances the proliferation and metastasis of CRC cells both in vivo and in vitro. A) LLGL2 expression was analyzed in different CRC cells. B) Western blotting analysis of the LLGL2 knockdown efficiency in SW620 cells. C) The CCK8 assay was employed to assess the proliferation of SW620 cells with LLGL2 knockdown. D) The colony formation assay was used to evaluate the proliferation of SW620 cells following LLGL2 knockdown. E) The migration ability of SW620 cells with LLGL2 knockdown was detected by scratch assay. F) Vim, E‐cad and N‐cad protein expression changes were analyzed in SW620 cells after LLGL2 knockdown. G) Subcutaneous tumors in shNC and shLLGL2 group. H) Volume variations during tumor growth in shNC and shLLGL2 groups. I) Statistics of subcutaneous tumor weight in shNC and shLLGL2 groups. (n = 6 per group). J) Ki67 IHC staining of subcutaneous tumors in shNC and shLLGL2 groups. Scale bar, 50 µm. K) Statistics regarding the quantity of liver metastasis tumor nodules in shNC and shLLGL2 groups (n = 5 per group). L) The liver‐to‐body weight ratio of mice in shNC and shLLGL2 groups (n = 5 per group). M) Liver metastasis tumors are provided in shNC and shLLGL2 groups, along with HE staining. Scale bar, 2000 µm. N) Statistics regarding the quantity of lung metastasis tumor nodules in shNC and shLLGL2 groups (n = 6 per group). O) Lung metastasis tumors are provided in shNC and shLLGL2 groups, along with HE staining. Scale bar, 1000 µm. P) The lung‐to‐body weight ratio of mice in shNC and shLLGL2 groups (n = 6 per group). Q) Structural representation of HL and predicted binding mode with LLGL2. Data are presented as mean ± SD. *P*‐values are determined by a two‐tailed Student's *t*‐test or one/two‐way ANOVA. *P< 0.05, **P < 0.01, ***P < 0.001.

Based on earlier research, we examined the functional significance of LLGL2 in CRC cells. We assessed the LLGL2 expression levels in various CRC cell lines. For subsequent experiments, we selected the SW620 cells, which exhibited higher LLGL2 expression, and RKO cells, which exhibited low expression (**Figure** **2**A). SW620 cells were transfected with short‐interfering RNA to induce endogenous LLGL2 knockdown (Figure 2B). Concurrently, we ectopically expressed LLGL2 in RKO cells (Figure S1A, Supporting Information). The Cell Counting Kit‐8 (CCK8) assay indicated that LLGL2 deficiency promoted SW620 cell proliferation (Figure 2C), whereas the overexpression of LLGL2 inhibited the growth of RKO cells (Figure S1B, Supporting Information). Moreover, LLGL2 knockdown increased SW620 cell clonogenesis (Figure 2D) and its overexpression had the opposite impact (Figure S1C, Supporting Information). Additionally, scratch tests showed that LLGL2 knockdown increased SW620 cell migration (Figure 2E), whereas its overexpression decreased RKO cells' migration (Figure S1D, Supporting Information). Immunofluorescence (IF) experiments showed that LLGL2 overexpression increased the fluorescence signal of E‐cadherin (E ‐ cad） in SW620 cells (Figure S1E, Supporting Information). Furthermore, LLGL2 knockdown enhanced the expression of Vimentin (Vim) and N‐cadherin (N‐cad) while inhibiting that of E‐cadherin (Figure 2F), whereas LLGL2 overexpression leads to the opposite result (Figure S1F, supporting information). These data suggested that LLGL2 is critical for controlling the EMT capacity of CRC cells. Next, we performed allogeneic transplantation experiments in BALB/c mice by subcutaneously injecting LLGL2 knockdown CT26 cells. The results demonstrated that the LLGL2 deficiency promoted tumor growth, resulting in increased tumor volume (Figure 2G,H) and weight (Figure 2I). Correspondingly, staining for the cell proliferation marker, Ki67, was elevated in LLGL2‐deficient tumors (Figure 2J). To further investigate the impact of LLGL2 on the in vivo metastasis of CRC cells, we established liver and lung metastasis models by injecting CT26 cells into the spleen and tail veins, respectively. LLGL2 deficiency significantly enhanced the dissemination of CRC cells to the liver, resulting in an increased number of liver metastatic nodules and a higher liver‐to‐body weight ratio (Figure 2K–M). Moreover, the lung metastasis model corroborated these findings (Figure 2N–P). In contrast, the overexpression of LLGL2 led to a decrease in the growth rate of subcutaneous tumors in mice (Figure S1G–I, Supporting Information). The mouse liver metastasis model also indicated that the overexpression of LLGL2 inhibited the metastasis of CRC cells to the mouse liver (Figure S1J–L, Supporting Information) and the lung metastasis model showed consistent results (Figure S1M—O, Supporting Information). These experiments indicate that LLGL2 plays a critical role in the progression of CRC, and restoring LLGL2 expression can inhibit the development of CRC. Numerous studies have demonstrated that natural products possess remarkable efficacy in suppressing tumor growth through multiple mechanisms.^[^ ^26^, ^27^ ^]^ To further explore whether activating LLGL2 expression has therapeutic potential for CRC, we discovered that Harmalol (HL), the main active component in the natural product *Peganum harmala* L., has the potential to bind to the LLGL2 protein (Figure 2Q). To evaluate the therapeutic potential of HL, we conducted a series of animal experiments for validation. The results showed that HL significantly inhibited tumor growth in vivo (Figure S7A—C, Supporting Information) and upregulated LLGL2 expression while inhibiting PI3K‐Akt activation (Figure S7D, Supporting Information). HL also mitigated the metastasis of CRC to the liver (Figure S7E—G, Supporting Information) and lungs (Figure S7H—J, Supporting Information). Overall, these results establish that HL can inhibit the progression of CRC by activating the expression of LLGL2.

### 2.3 Adeno‐Associated Virus (AAV)‐Mediated LLGL2 Knockdown Promotes the Development of AOM/DSS‐Induced CRC

**Figure 3:**
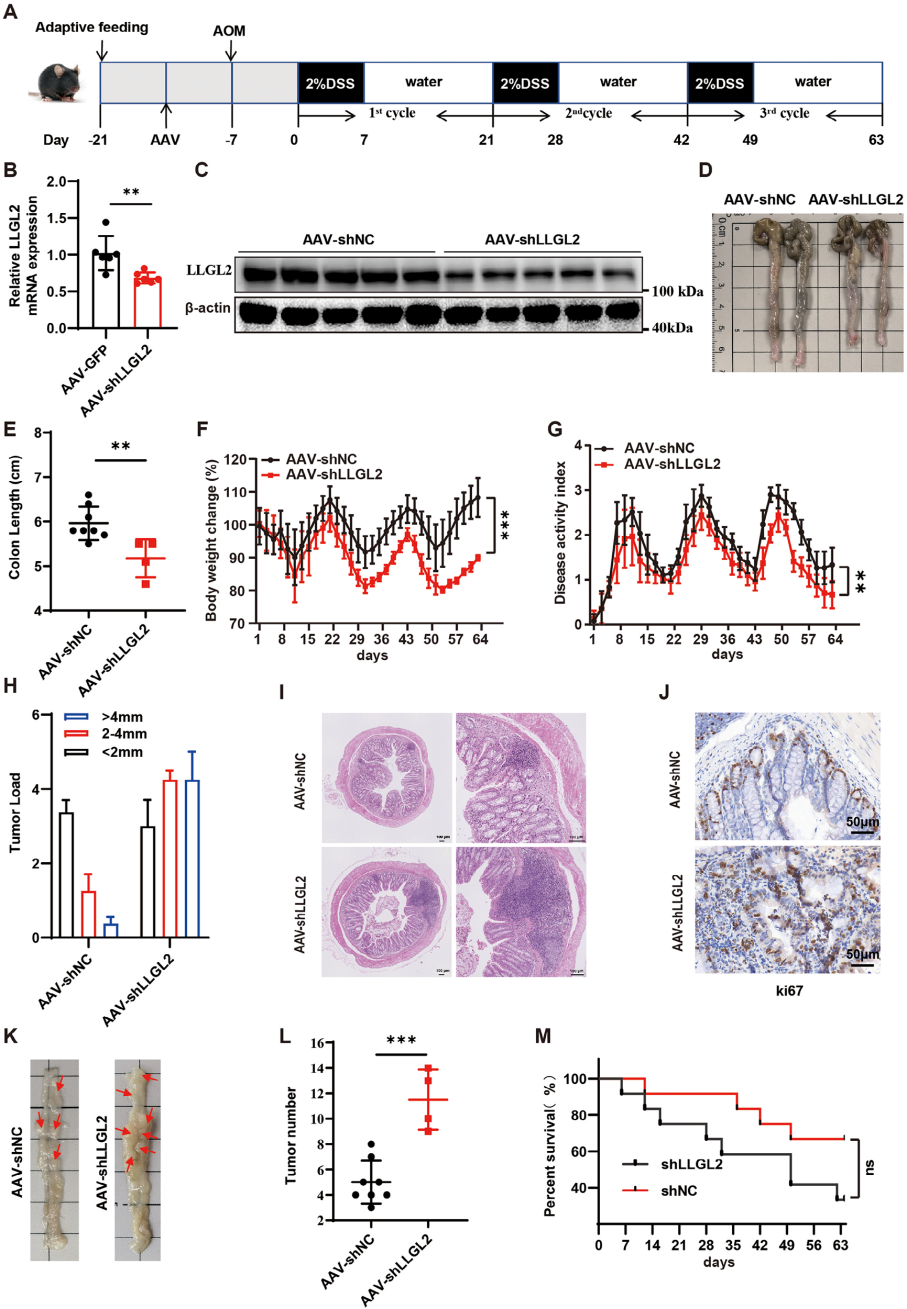
AAV‐mediated LLGL2 knockdown promotes the development of AOM/DSS‐induced CRC. A) Schematic representation of the CRC model induced by AOM/DSS. B) RT‐qPCR analysis of LLGL2 expression in colon of mice 5 weeks after injection of AAV‐shNC and AAV‐shLLGL2 without AOM/DSS treatment (n = 6 per group). C) LLGL2 expression was analyzed in colon of mice 4 weeks after injection of AAV‐shNC and AAV‐shLLGL2 without AOM/DSS treatment (n = 5 per group). D) Colon images representing the AAV‐shNC and AAV‐shLLGL2 groups. E) Statistics of colon length of mice in AAV‐shNC and AAV‐shLLGL2 groups. F,G) Body weight changes and DAI in BALB/c mice with AOM/DSS induced CRC were monitored every 2 days in the AAV‐shNC group and the AAV‐shLLGL2 group (AAV‐shNC, n = 8, AAV‐shLLGL2, n = 4). H) Data on tumor size distribution relative to tumor numbers (tumor load) were collected. I) HE staining of AAV‐shNC and AAV‐shLLGL2 mouse colons were shown scale bar, 100 µm. J) Ki67 stained of mouse colon from the AAV‐shNC and AAV‐shLLGL2 groups. Scale bar, 50 µm. K) Colon tumor in AAV‐shNC and AAV‐shLLGL2 mice were shown. L) Statistics on colon tumor counts in AAV‐shNC and AAV‐shLLGL2 groups (AAV‐shNC n = 8, AAV‐shLLGL2, n = 4). M) Survival rate statistics of mice in AAV‐shNC and AAV‐shLLGL2 groups. based on the log‐rank test. Data are presented as mean ± SD. *P*‐values are determined by a two‐tailed Student's *t*‐test. **P < 0.01, ***P < 0.001, ns (not significant).

To understand the role of LLGL2 at the beginning of CRC, we established an AOM/DSS‐induced mouse CRC model (**Figure** **3**A). Recombinant AAV vector‐mediated gene delivery to the intestinal epithelial cells is a novel method for gut transduction that facilitates the study of intestinal diseases.^[^ ^28^ ^]^ We chose AAV serotype 9, which is known to demonstrate comparatively high efficacy in gut transduction.^[^ ^29^ ^]^ AAV for LLGL2 knockdown (AAV‐shLLGL2) or control vectors (AAV‐shNC) were then administered by rectal injection to male BALB/c mice that were five weeks old. To assess the efficiency of intestinal transduction in vivo, we performed western blotting and quantitative reverse transcription PCR (RT‐qPCR) to evaluate LLGL2 expression in the colon (Figure 3B,C). In the AOM/DSS‐induced CRC model, BALB/c mice treated with AAV‐shLLGL2 exhibited significantly shorter colon lengths (Figure 3D,E), greater weight loss (Figure 3F), a higher disease activity index (DAI) (Figure 3G), larger tumor volumes (Figure 3H,I), and an increased number of colon tumors (Figure 3K,L) compared with those of AAV‐shNC mice. Furthermore, higher Ki67 expression was observed in the colon tissues of LLGL2‐deficient mice (Figure 3J). In addition, LLGL2‐deficient mice demonstrated lower survival rates; however, the difference was not statistically significant (Figure 3M). Overall, these findings implied that AAV‐mediated silencing of LLGL2 in the colon accelerated CRC development in mice.

### 2.4 LLGL2 Binds to THBS3 mRNA and Affects its Stability

**Figure 4:**
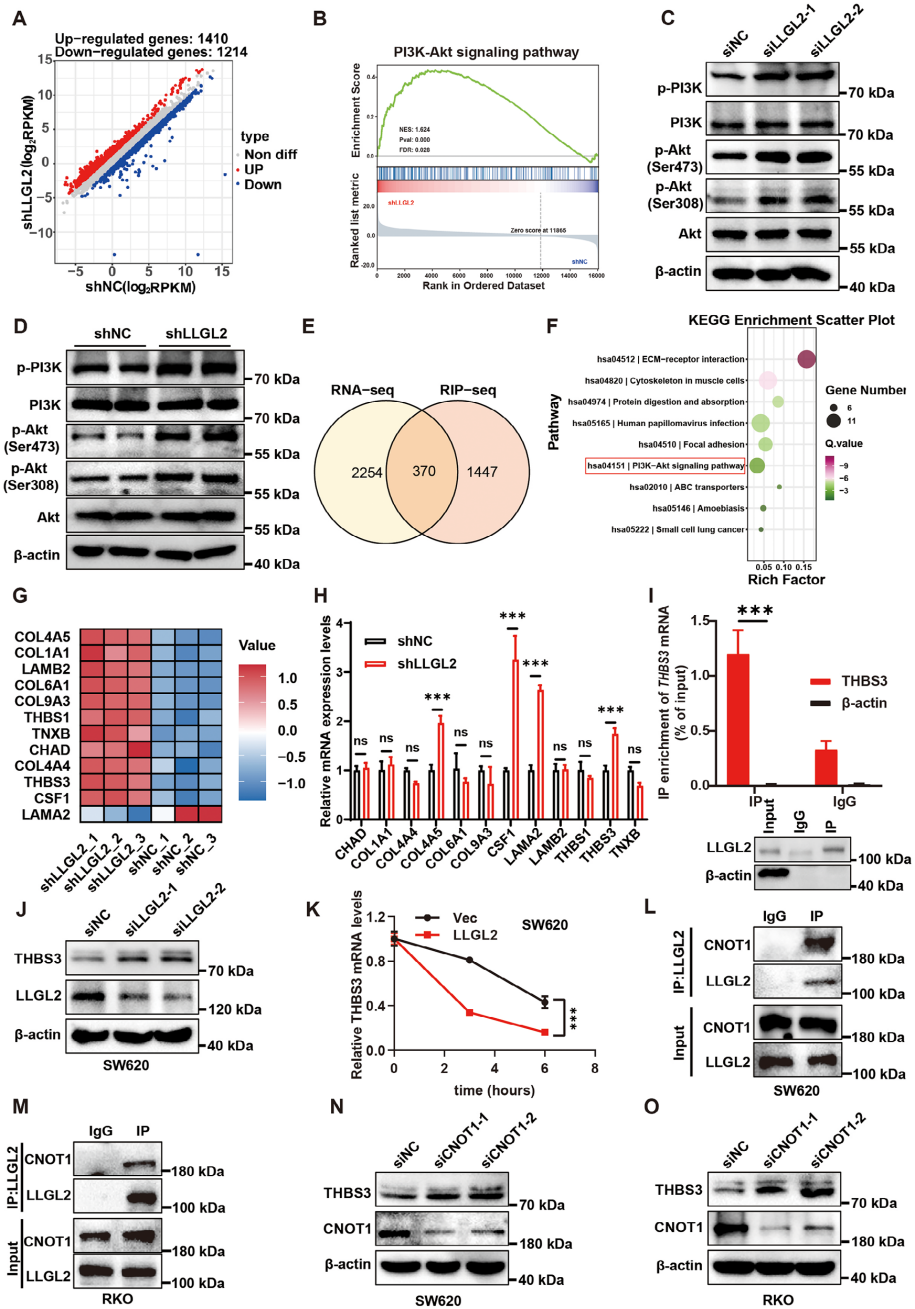
LLGL2 binds to *THBS3* mRNA and affects its stability. A) Scatter plot showing all expression changes of genes in SW620 cells expressing shNC or shLLGL2. B) All differential genes after LLGL2 knockdown in SW620 cells were analyzed by GSEA. C) The protein alterations of p‐PI3K and p‐Akt were analyzed following LLGL2 knockdown in SW620 cells. D) The p‐PI3K and p‐Akt in subcutaneous tumor tissues of shNC and shLLGL2 mice were analyzed. E) Venn diagram of RNA‐seq and RIP‐seq showing that the mRNA of 370 genes was bound and regulated by LLGL2. F) KEGG analysis of 370 overlapping genes. G) Expression heat map of 12 genes related to PI3K‐Akt pathway in RNA‐seq results. H) RT‐qPCR analysis of 12 PI3K‐Akt pathway genes following LLGL2 knockdown in SW620 cells. I) RIP‐qPCR analysis of endogenous LLGL2 and *THBS3* mRNA binding in SW620 cells. J) Analysis of THBS3 protein expression changes after LLGL2 knockdown in SW620 cells. K) RT‐qPCR analysis of *THBS3* mRNA half‐life alterations after LLGL2 overexpression in SW620 cells. L) LLGL2‐CNOT1 interaction in SW620 cells. Co‐IP assays using anti‐LLGL2 (IP) versus IgG control. M) LLGL2‐CNOT1 interaction in RKO cells. Co‐IP assays using anti‐LLGL2 (IP) versus IgG control. N) Western blotting experiment analyzed changes in the protein levels of THBS3 after knocking down CNOT1 in SW620 cells. O) Western blotting experiment analyzed changes in the protein levels of THBS3 after knocking down CNOT1 in RKO cells. Data are presented as mean ± SD. *P*‐values are determined by a two‐tailed Student's *t*‐test or two‐way ANOVA., ***P < 0.001, ns (not significant).

To probe evens downstream of the reduced LLGL2 expression, we conducted RNA‐seq on SW620 cells with knocked‐down LLGL2. As a result, 1214 RNAs were downregulated and 1410 RNAs were upregulated (**Figure** **4**A). Gene set enrichment analysis (GSEA) indicated that the PI3K‐Akt signaling pathway was one of the primary pathways activated following LLGL2 knockdown (Figure 4B). Subsequently, we observed a significant activation of phosphorylated PI3K (p‐PI3K) and enhanced phosphorylation of Akt at serine 308 (Ser308) and serine 473 (Ser473) in LLGL2‐knockdown SW620 cells (Figure 4C). Conversely, LLGL2 overexpression in RKO cells markedly inhibited the phosphorylation of Akt and PI3K (Figure S2A, Supporting Information). In the subcutaneous tumor tissues of the mice, we observed an increase in the phosphorylation of Akt and PI3K following the knockdown of LLGL2 (Figure 4D). This result was further validated in tumors overexpressing LLGL2 (Figure S2B, Supporting Information), indicating that a reduction in LLGL2 expression is a key factor in promoting the progression of CRC. Previous reports have indicated that LLGL2 functions as an RBP. To explore whether LLGL2 acts as an RBP in CRC, we conducted RIP‐seq experiments to identify RNAs that directly bind to LLGL2. Through intersectional analysis of RNA‐seq data, we identified 370 candidate RNAs that may both bind to and be regulated by LLGL2 (Figure 4E). KEGG analysis results revealed that the PI3K‐Akt pathway was the main downstream pathway effected by RNA binding to LLGL2 (Figure 4F). Among these, 12 RNAs were primarily associated with the PI3K‐Akt pathway (Figure 4G). We verified the transcription levels of these 12 genes in cells and found that only *THBS3* mRNA expression showed a consistent trend in both LLGL2‐knockdown SW620 cells and LLGL2‐overexpressed RKO cells (Figure 4H; Figure S2C, Supporting Information). Finally, the RIP‐qPCR assay demonstrated an interaction between *THBS3* mRNA and LLGL2 proteins (Figure 4I; Figure S2D, Supporting Information). Western blotting indicated that knocking down LLGL2 increased the protein expression of THBS3 (Figure 4J), whereas overexpression of LLGL2 inhibited THBS3 protein expression (Figure S2E, Supporting Information). Subsequently, actinomycin D was added to assess the half‐life of *THBS3* mRNA. LLGL2 overexpression significantly reduced the stability of *THBS3* mRNA (Figure 4K; Figure S2F, Supporting Information). We suspected that LLGL2 might regulate the stability of *THBS3*. Given the absence of reports on the role of LLGL2 in RNA stability, we conducted shotgun mass spectrometry analysis to determine whether proteins interacting with LLGL2 influence the stability of *THBS3* mRNA. Our analysis revealed that CNOT1 interacts with LLGL2 (Figure S2G, Supporting Information). CNOT1 functions as a framework for RBPs, attracting the CCR4‐NOT complex to the 3ʹ‐UTR of target mRNAs, which leads to their degradation.^[^ ^30^, ^31^ ^]^ Using co‐immunoprecipitation (Co‐IP), we demonstrated that LLGL2 interacted with CNOT1 (Figure 4L,M; Figure S2H,I, Supporting Information), and the knockdown of CNOT1 promoted the protein expression of THBS3 (Figure 4N,O). This suggests that LLGL2 may affect the stability of *THBS3* by interacting with CNOT1, thereby regulating THBS3 expression. The N‐terminus of LLGL2 contains multiple WD40 repeat domains that scaffold RNA interactions, functioning as an RBP in tumors by binding the lncRNA MAYA via its first three WD40 domains to activate YAP signaling and promote bone metastasis.^[^ ^19^, ^20^, ^21^ ^]^This suggests that LLGL2 functions as an RBP in tumors. To identify the critical region of LLGL2 that interacts with *THBS3* mRNA, we constructed two deletion mutants of LLGL2: one at the N‐terminus and the other at the C‐terminus (Figure S2J, Supporting Information). RIP‐qPCR experiments demonstrated that the N‐terminus domain of LLGL2 served as the primary active region responsible for binding to *THBS3* mRNA(Figure S2K, Supporting Information). Additionally, rescue experiments indicated that overexpression of CNOT1 could partially reverse the increase in THBS3 protein levels caused by LLGL2 knockdown (Figure S2L, Supporting Information). In brief, the expression of LLGL2 protein, through its binding to *THBS3* mRNA, and the subsequent reduction in its stability, may represent a key intermediate molecular event in the activation of the PI3K‐Akt pathway induced by LLGL2 knockdown.

### 2.5 THBS3 Knockdown Reduces CRC Cell Proliferation and Metastasis, and Lower THBS3 Levels Correlate with Prolonged Survival

**Figure 5:**
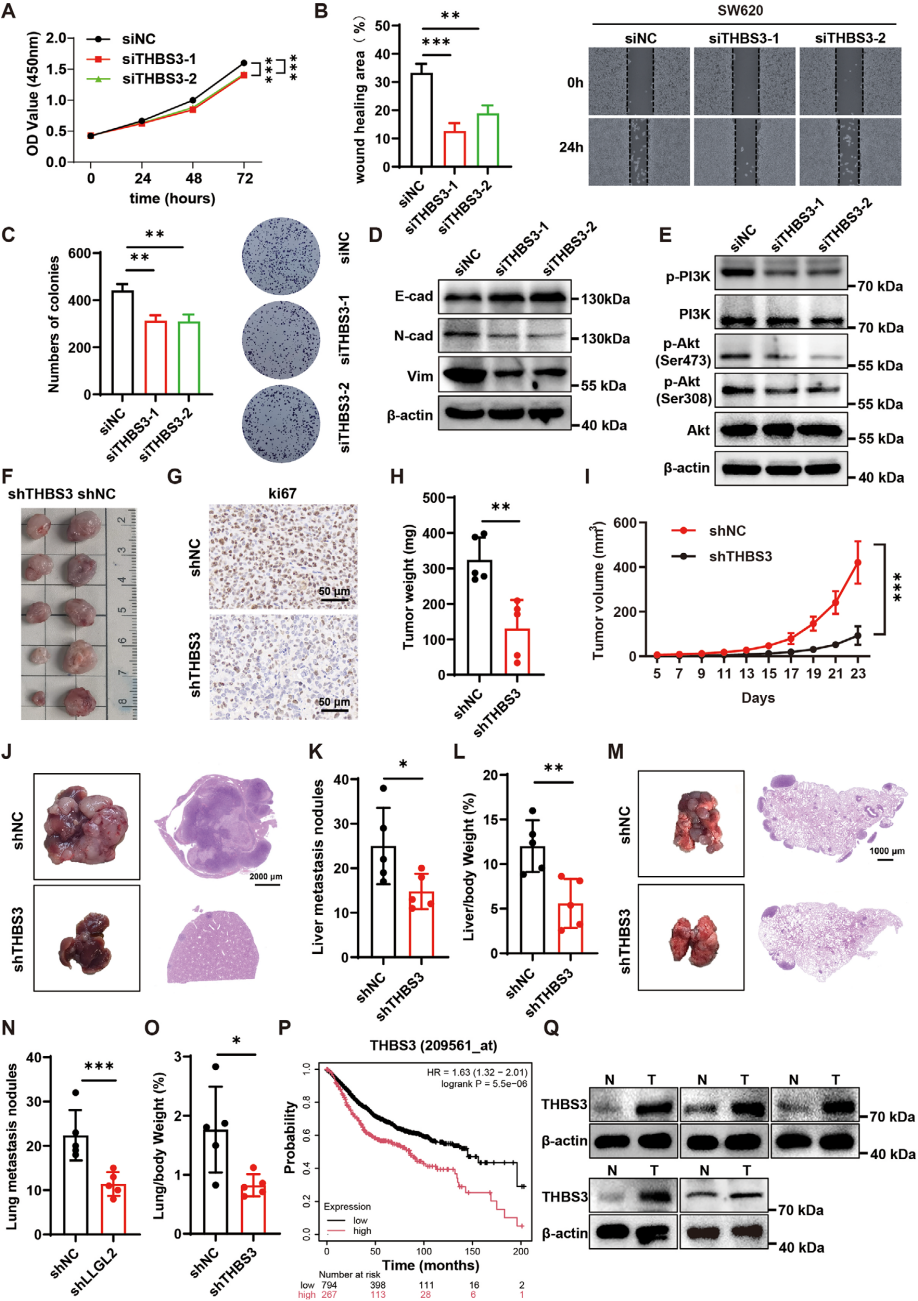
THBS3 knockdown reduces CRC cell proliferation and metastasis, and lower THBS3 levels correlate with longer survival. A) CCK‐8 proliferation assay of THBS3‐knockdown SW620 cells. B) Wound healing assay of THBS3‐knockdown SW620 cells. C) Colony formation assay of THBS3‐knockdown SW620 cells. D) Western blotting analysis of EMT markers in THBS3‐knockdown SW620 cells. E) The protein expression of p‐PI3K and p‐Akt were analyzed in SW620 cells with THBS3 knockdown. F) Subcutaneous tumors in the shNC group and the shTHBS3 group were shown. G) Ki67 IHC staining of subcutaneous tumors in the shNC group and the shTHBS3 group. scale bar, 50 µm. H) Statistics of subcutaneous tumor weight in the shNC group and the shTHBS3 group (n = 5 per group). I) Volume variations during tumor growth in shNC and shLLGL2 groups. J) Representative images of liver metastasis tumors are provided in the shNC group and the shTHBS3 group, along with HE staining. Scale bar, 2000 µm. K) Statistics regarding the quantity of liver metastasis tumor nodules in the shNC group and the shTHBS3 group. (n = 5 per group). L) The liver‐to‐body weight ratio of mice in shNC and shLLGL2 groups (n = 5 per group). M) Lung metastasis tumors are provided in the shNC group and the shTHBS3 group, along with HE staining. scale bar, 1000 µm. N) Statistics regarding the quantity of lung metastasis tumor nodules in the shNC group and the shTHBS3 group. O) The lung‐to‐body weight ratio of mice in shNC and shTHBS3 groups. P) Kaplan–Meier analysis of OS in patients with high versus low THBS3 expression Q) The THBS3 expression were analyzed in 5 pairs of CRC tissues from patient. (N for non‐tumor tissue, T for tumor tissue). Data are presented as mean ± SD. *P*‐values are determined by a two‐tailed Student's *t*‐test or one/two‐way ANOVA. *P < 0.05, **P < 0.01, ***P < 0.001.THBS3 knockdown reduces CRC cell proliferation and metastasis, and lower THBS3 levels correlate with longer survival. A) CCK‐8 proliferation assay of THBS3‐knockdown SW620 cells. B) Wound healing assay of THBS3‐knockdown SW620 cells. C) Colony formation assay of THBS3‐knockdown SW620 cells. D) Western blotting analysis of EMT markers in THBS3‐knockdown SW620 cells. E) The protein expression of p‐PI3K and p‐Akt were analyzed in SW620 cells with THBS3 knockdown. F) Subcutaneous tumors in the shNC group and the shTHBS3 group were shown. G) Ki67 IHC staining of subcutaneous tumors in the shNC group and the shTHBS3 group. scale bar, 50 µm. H) Statistics of subcutaneous tumor weight in the shNC group and the shTHBS3 group (n = 5 per group). I) Volume variations during tumor growth in shNC and shLLGL2 groups. J) Representative images of liver metastasis tumors are provided in the shNC group and the shTHBS3 group, along with HE staining. Scale bar, 2000 µm. K) Statistics regarding the quantity of liver metastasis tumor nodules in the shNC group and the shTHBS3 group. (n = 5 per group). L) The liver‐to‐body weight ratio of mice in shNC and shLLGL2 groups (n = 5 per group). M) Lung metastasis tumors are provided in the shNC group and the shTHBS3 group, along with HE staining. scale bar, 1000 µm. N) Statistics regarding the quantity of lung metastasis tumor nodules in the shNC group and the shTHBS3 group. O) The lung‐to‐body weight ratio of mice in shNC and shTHBS3 groups. P) Kaplan–Meier analysis of OS in patients with high versus low THBS3 expression Q) The THBS3 expression were analyzed in 5 pairs of CRC tissues from patient. (N for non‐tumor tissue, T for tumor tissue). Data are presented as mean ± SD. *P*‐values are determined by a two‐tailed Student's *t*‐test or one/two‐way ANOVA. *P < 0.05, **P < 0.01, ***P < 0.001.

To gain a better understanding of how THBS3 functions in CRC, we knocked down its expression in SW620 cells (Figure S3A, Supporting Information). The migratory and proliferative capacities of SW620 cells were markedly inhibited by THBS3 knockdown (**Figure** **5**A–C). Additionally, the EMT capability associated with CRC was also reduced (Figure 5D), which was further confirmed by RT‐qPCR (Figure S3B, Supporting Information). Moreover, we observed a substantial decrease in the phosphorylation of PI3K and Akt (Figure 5E). In vivo experiments demonstrated that THBS3 deficiency slowed subcutaneous tumor growth, reduced tumor weight, and weakened proliferation in mice (Figure 5F–I). In addition, the mouse models of lung and liver metastases further indicated that THBS3 knockdown suppressed the metastatic spread of CRC cells to distal tissues (Figure 5J–O). Conversely, overexpression of THBS3 in RKO cells led to significant enhancements in proliferation (Figure S3C,D, Supporting Information), migration (Figure S3E, Supporting Information), and EMT capabilities (Figure S3F,G, Supporting Information), and increased levels of phosphorylated PI3K and Akt (Figure S3H, Supporting Information). According to the Kaplan–Meier curves, patients with high THBS3 expression levels had lower OS and RFS compared with that of low THBS3 expression (Figure 5P; Figure S3I, Supporting Information). Furthermore, western blotting revealed increased THBS3 expression in the tumor tissues of patients with CRC (Figure 5Q).

### 2.6 LLGL2 Impacts the Progression of CRC through Modulating the PI3K‐Akt Pathway via THBS3

**Figure 6:**
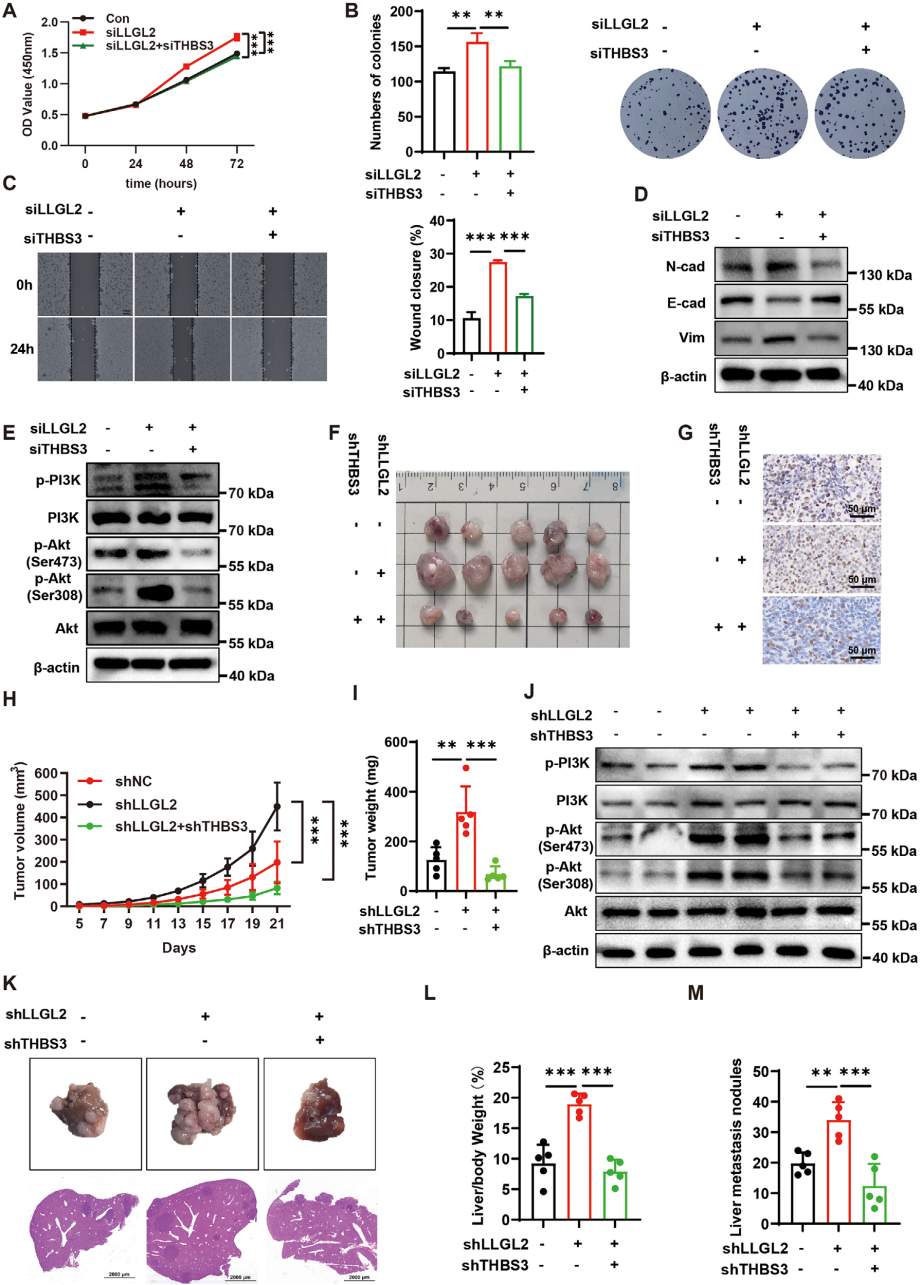
LLGL2 impacts the progression of CRC through modulating the PI3K‐Akt pathway via THBS3. A) CCK‐8 assay analyzing proliferation of SW620 cells after LLGL2 knockdown or combined LLGL2/THBS3 knockdown. B) Colony formation assay of SW620 cells after LLGL2 single or LLGL2/THBS3 double knockdown C) Wound healing assay of SW620 cells after LLGL2 knockdown or combined LLGL2/THBS3 knockdown. D) Western blotting analysis of EMT markers in LLGL2 and/or THBS3 knockdown SW620 cells. E) Western blotting analysis of PI3K‐Akt activation in LLGL2 and/or THBS3 knockdown SW620 cells. F) Representative images of subcutaneous tumors in control group, LLGL2 knockdown alone group, and LLGL2 combined with THBS3 knockdown group (n = 5). G) Representative Ki67 IHC staining image of subcutaneous tumors in different groups. scale, 50 µm. H) Tumor volume changes across differient groups. I) Subcutaneous tumor weight statistics of different groups (n = 5). J) Western blotting analysis of the protein levels of p‐PI3K and p‐Akt in different groups. K) Representative images of liver tumors across different groups. L) Statistical analysis of liver‐to‐body weight ratios across different groups (n = 5). M) Statistical analysis of the liver metastasis nodules among different groups (n = 5). Data are presented as mean ± SD. *P*‐values are determined by one/two‐way ANOVA. **P < 0.01, ***P < 0.001.LLGL2 impacts the progression of CRC through modulating the PI3K‐Akt pathway via THBS3. A) CCK‐8 assay analyzing proliferation of SW620 cells after LLGL2 knockdown or combined LLGL2/THBS3 knockdown. B) Colony formation assay of SW620 cells after LLGL2 single or LLGL2/THBS3 double knockdown C) Wound healing assay of SW620 cells after LLGL2 knockdown or combined LLGL2/THBS3 knockdown. D) Western blotting analysis of EMT markers in LLGL2 and/or THBS3 knockdown SW620 cells. E) Western blotting analysis of PI3K‐Akt activation in LLGL2 and/or THBS3 knockdown SW620 cells. F) Representative images of subcutaneous tumors in control group, LLGL2 knockdown alone group, and LLGL2 combined with THBS3 knockdown group (n = 5). G) Representative Ki67 IHC staining image of subcutaneous tumors in different groups. scale, 50 µm. H) Tumor volume changes across differient groups. I) Subcutaneous tumor weight statistics of different groups (n = 5). J) Western blotting analysis of the protein levels of p‐PI3K and p‐Akt in different groups. K) Representative images of liver tumors across different groups. L) Statistical analysis of liver‐to‐body weight ratios across different groups (n = 5). M) Statistical analysis of the liver metastasis nodules among different groups (n = 5). Data are presented as mean ± SD. *P*‐values are determined by one/two‐way ANOVA. **P < 0.01, ***P < 0.001.

Subsequently, we conducted a rescue experiment in which simultaneous knockdown of LLGL2 and THBS3 in SW620 cells partially reversed the increased proliferation (**Figure** **6**A,B), migration (Figure 6C), and EMT capabilities (Figure 6D) induced by LLGL2 knockdown alone. This dual knockdown also inhibited the increase in p‐PI3K and p‐Akt levels caused by LLGL2 knockdown (Figure 6E). These conclusions were further substantiated in a mouse model of subcutaneous tumors. Knockdown of both LLGL2 and THBS3 can partially counteracted the increase in tumor growth and proliferation caused by the knockdown of LLGL2 alone (Figure 6F–I). Additionally, stimulation of the PI3K‐Akt pathway induced by LLGL2 knockdown alone was partially reversed by the combined knockdown (Figure 6J). Furthermore, THBS3 knockdown inhibited the exacerbation of liver metastasis in CRC induced by the knockdown of LLGL2 (Figure 6K–M). A similar conclusion was reached in the lung metastasis model (Figure S4A–C, Supporting Information). Similarly, the simultaneous overexpression of LLGL2 and THBS3 in RKO cells partially reversed the decreased proliferation (Figure S4D,E, Supporting Information), migration (Figure S4F, Supporting Information), and EMT capabilities (Figure S4G, Supporting Information) that resulted from LLGL2 overexpression alone and also partially restored the reduced expression levels of p‐PI3K and p‐Akt (Figure S4H, Supporting Information). These results suggested that reduced LLGL2 expression facilitates CRC progression by elevating THBS3 expression. This elevation potentially acts as a molecular connection that enables LLGL2 to activate the PI3K‐Akt pathway.

### 2.7 MDM2 Engages in Interaction with LLGL2 and Modulates Its Levels

**Figure 7:**
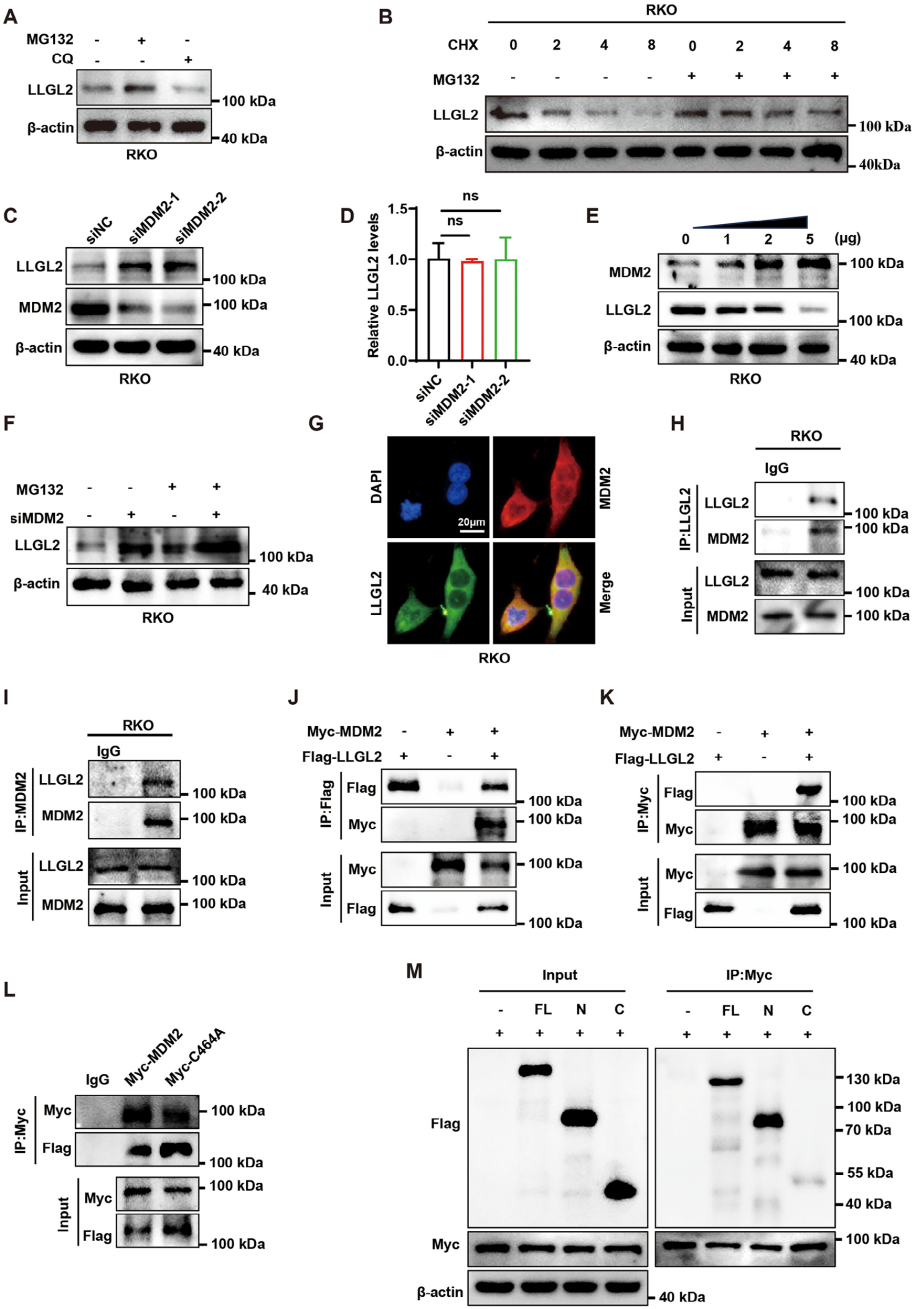
MDM2 engages in interaction with LLGL2 and modulates its levels. A) Analysis of LLGL2 protein in RKO cells post‐treatment with 10 µM MG132 or 20 µM CQ. B) Analysis of LLGL2 protein changes in RKO cells treated with 50 µg mL^−1^ CHX for varying durations, with or without 10 µM MG132. C) Western blotting analysis of LLGL2 protein expression in MDM2‐knockdown RKO cells. D) RT‐qPCR analysis of LLGL2 expression in MDM2‐knockdown RKO cells. E) LLGL2 protein levels in RKO cells following transfection with varying doses of MDM2 plasmid. F) LLGL2 protein levels in RKO cells following the knockdown of MDM2 expression, with and without treatment with 10 µM MG132. G) Colocalization of MDM2 and LLGL2 in RKO cells by IF. H,I) Co‐IP analysis of endogenous MDM2 and LLGL2 interaction in RKO cells. J,K) Flag‐LLGL2 and Myc‐MDM2 were transfected into HEK293T cells, and a Co‐IP assay was conducted to examine the interaction between Flag‐LLGL2 and Myc‐MDM2. L) Flag‐LLGL2, Myc‐MDM2 and Myc‐C464A were transfected into HEK293T cells, and Co‐IP assay was used to analyze the interaction between Flag‐LLGL2 and Myc‐MDM2 or Myc‐C464A. M) Analysis of the interaction between MDM2 and truncation mutants of LLGL2 in HEK293T cells. Data are presented as mean ± SD. *P*‐values are determined by one‐way ANOVA. ns (not significant).

The CPTAC database results showed that the tumor tissues of patients with CRC had a considerably lower levels of LLGL2 protein expression. In contrast, TCGA database showed that these patients' tumor and para‐cancerous tissues did not express different amounts of *LLGL2* mRNA. This discrepancy suggests that post‐translational regulation influences LLGL2 protein levels. To investigate the potential for proteasomal or lysosomal degradation of LLGL2, we treated CRC cells with 10 µM of the proteasome inhibitor MG132 and 20 µM of the lysosomal inhibitor chloroquine (CQ). Exposure to MG132 substantially increased LLGL2 protein expression, whereas CQ treatment had little effect on LLGL2 protein levels (**Figure** **7**A; Figure S5A, Supporting Information). Subsequent cycloheximide (CHX) chase experiments demonstrated that MG132 treatment effectively mitigated CHX‐induced reduction in LLGL2 protein expression (Figure 7B; Figure S5B, Supporting Information). These findings suggest that LLGL2 may be degraded via the ubiquitin‐proteasome system. We used Ubibrowser (http://ubibrowser.ncpsb.org.cn/Ubibrowser/) to predict E3 ubiquitin ligases (Figure S5C, Supporting Information).^[^ ^32^ ^]^ Among the top ten predicted E3 ubiquitin ligases predicted, we found a clear negative correlation between MDM2 and LLGL2 expression levels in CRC (Figure S5D, Supporting Information). Subsequently, we investigated the possible role of MDM2 as a factor upstream of LLGL2 in regulating its expression. In the present study, we silenced MDM2 in RKO cells, which was accompanied by a corresponding increase in LLGL2 expression (Figure 7C). Similarly, in SW620 cells, LLGL2 protein levels decreased upon MDM2 overexpression (Figure S5E, Supporting Information). However, the transcript levels of LLGL2 remained unchanged regardless of MDM2 overexpression or knockdown (Figure 7D; Figure S5F, Supporting Information). Notably, as MDM2 overexpression increased, the reduction in LLGL2 protein expression became more pronounced (Figure 7E; Figure S3G, Supporting Information). MG132 treatment further enhanced LLGL2 expression resulting from the knockdown of MDM2 in RKO cells (Figure 7F). Similarly, MG132 treatment partially mitigated the reduction in LLGL2 expression induced by the overexpression of MDM2 in SW620 cells (Figure S5H, Supporting Information). IF experiments confirmed the colocalization of MDM2 and LLGL2 (Figure 7G; Figure S5I, Supporting Information). We further explored the interaction between endogenous MDM2 and LLGL2 using Co‐IP assays (Figure 7H,I; Figure S5J,K, Supporting Information). Subsequently, we transfected Myc‐MDM2 and Flag‐LLGL2 into HEK293T and SW620 cells and use the corresponding tag antibodies for Co‐IP and western blotting, which further substantiated the interaction between MDM2 and LLGL2 (Figure 7J,K; Figure S5L,M, Supporting Information). Notably, the enzymatic activity mutant, C464A of MDM2, did not affect its interaction with LLGL2 (Figure 7L). In addition, Co‐IP assays showed that the N‐terminus of LLGL2 could interact with MDM2, whereas the C‐terminus could not. (Figure 7M). These findings suggested that MDM2 negatively regulates LLGL2 protein expression via its interaction with LLGL2.

### 2.8 MDM2 Promotes the Ubiquitination and Degradation of LLGL2

**Figure 8:**
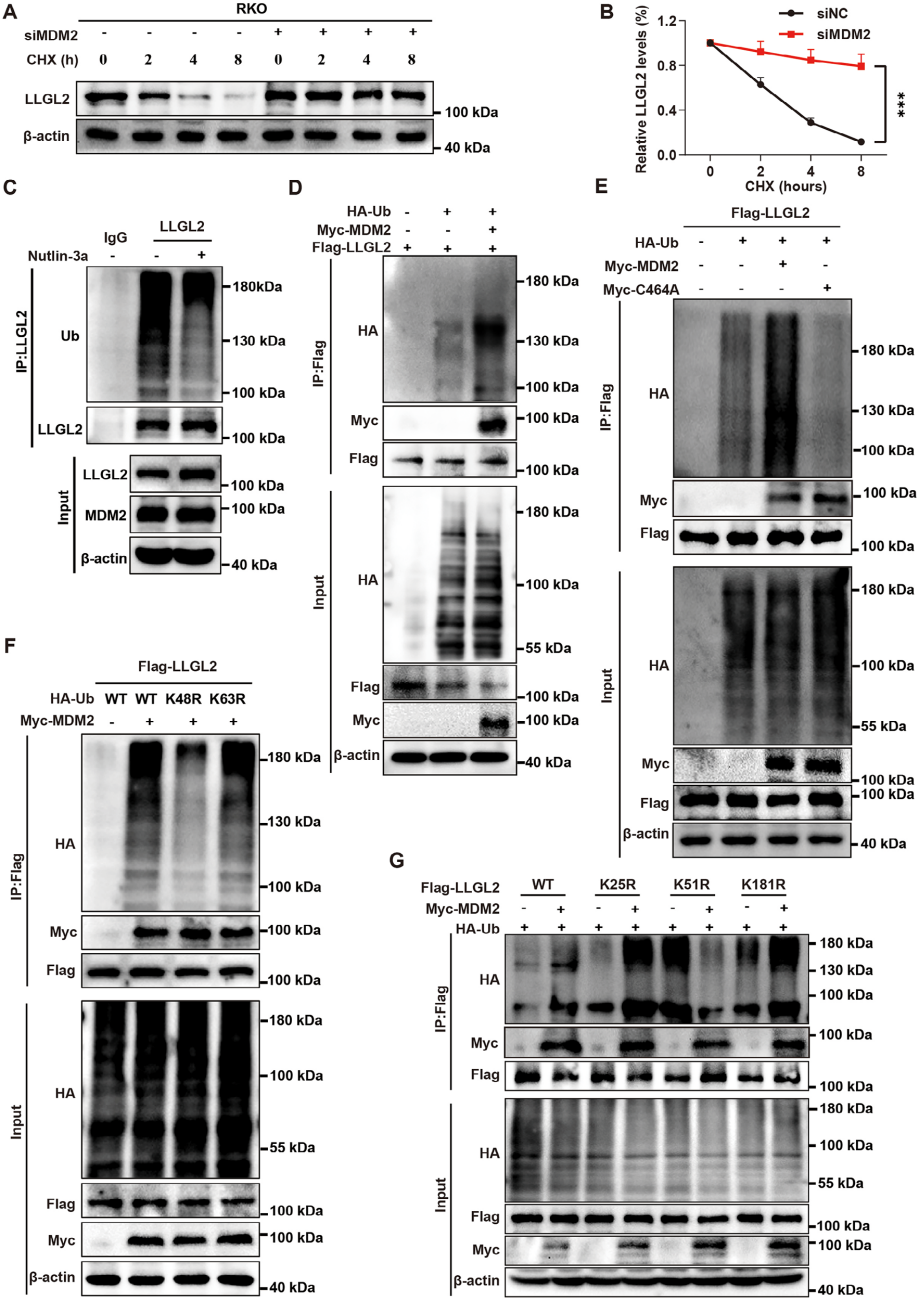
MDM2 promotes the ubiquitination and degradation of LLGL2. A) RKO cells were either transfected with or without siMDM2. Following treatment with CHX for a specified duration, the LLGL2 protein expression alterations were evaluated by western blotting. B) Quantification of LLGL2 protein in siNC and siMDM2 groups treated with CHX for indicated times. C) The endogenous ubiquitination level of LLGL2 in RKO cells treated with or without Nutlin‐3a. D) HEK293T cells were transfected with HA‐Ub, Myc‐MDM2 and Flag‐LLGL2 plasmids and then subjected to Co‐IP experiment, and the expression level of HA‐Ub was analyzed. E) HEK293T cells were transfected with HA‐Ub, Myc‐MDM2, Flag‐LLGL2 and Myc‐C464A plasmids and then Co‐IP experiments were performed. The expression level of HA‐Ub was used to analyze. F) HEK293T cells were co‐transfected with Myc‐MDM2, Flag‐LLGL2, HA‐Ub and its different mutants. Flag antibody was used for Co‐IP experiment, and the expression level of HA‐Ub was used to analyze. G) HEK293T cells were co‐transfected with HA‐Ub and various Flag‐LLGL2 mutates, with or without Myc‐MDM2 plasmid, and Co‐IP experiment was performed. The expression level of HA‐Ub was analyzed. Data are presented as mean ± SD. *P*‐values are determined by two‐way ANOVA. ***P < 0.001.

To further confirm that MDM2 facilitates the degradation of LLGL2 protein via ubiquitination, we conducted a CHX chasing experiment. Following the knockdown of MDM2 in RKO cells, the degradation half‐life of the LLGL2 protein was markedly extended (**Figure** **8**A,B). Conversely, the overexpression of MDM2 in SW620 cells significantly reduced the half‐life of LLGL2 degradation (Figure S6A,B, Supporting Information). Furthermore, the MDM2 enzyme‐active mutant, C464A, did not exert a significantly influence the degradation half‐life of LLGL2 (Figure S6C,D, Supporting Information). Next, we evaluated the effect of MDM2 on endogenous LLGL2 ubiquitination using the Co‐IP. Consistent with our hypothesis, the results indicated that, following MDM2 knockdown, the ubiquitination level of LLGL2 in RKO cells was significantly reduced, accompanied by an increase in LLGL2 protein expression (Figure S6E, Supporting Information). Similarly, in SW620 cells overexpressing MDM2, the ubiquitination level of LLGL2 was significantly elevated, which correlated with a decrease in LLGL2 protein expression (Figure S6F, Supporting Information). Furthermore, we treated RKO cells with Nutlin‐3a, a well‐characterized MDM2 inhibitor. The ubiquitination level of LLGL2 was markedly reduced, while its expression was significantly upregulated (Figure 8C). To further validate that MDM2 mediates the ubiquitination of LLGL2, we transfected HA‐Ub, Myc‐MDM2, and Flag‐LLGL2 plasmids into HEK293T cells. Co‐IP experiments using Flag antibodies demonstrated that exogenously introduced Myc‐MDM2 enhanced the ubiquitination of Flag‐LLGL2 (Figure 8D). This finding was corroborated in the SW620 cells (Figure S6G, Supporting Information). The enzymatic mutant of MDM2, Myc‐C464A, did not significantly affect the ubiquitination of LLGL2 (Figure 8E; Figure S6H, Supporting Information), implying that the enzymatic activity of MDM2 is important in mediating the ubiquitination of LLGL2. To determine the type of ubiquitination occurring in LLGL2, we co‐transfected Flag‐LLGL2, Myc‐MDM2 and HA‐tagged ubiquitin mutants, which mutated lysine at position 48 to arginine (K48R) or lysine at position 63 to arginine (K63R). Conversely, the K48R mutant showed reduced ubiquitination levels of LLGL2, suggesting that MDM2 primarily promotes K48‐linked ubiquitin chains in LLGL2 (Figure 8F). This finding was corroborated in the SW620 cells (Figure S6I, Supporting Information). We used a database (https://gpsuber.biocuckoo.cn/index.php) to predict lysine sites on the LLGL2 protein that may be subject to ubiquitination (Figure S6J, Supporting Information).^[^ ^33^ ^]^ Based on prediction scores, we focused on the three highest‐ranking lysine residues for mutagenesis analysis: lysine at position 25 (K25), lysine at position 51 (K51), and lysine at position 181 (K181). These candidate sites were individually mutated to arginine (K25R, K51R, and K181R) to assess their functional importance. Various mutant plasmids of LLGL2 were constructed and tagged with Flag tags. Each LLGL2 mutant was co‐transfected with HA‐Ub and Myc‐MDM2 into HEK293T cells. Notably, mutation of the K51 site resulted in a significant reduction in MDM2‐mediated ubiquitination (Figure 8G). Collectively, these findings clearly demonstrated that MDM2 is responsible for K48‐linked ubiquitination of LLGL2 at the K51 site.

To further validate our findings in vivo, we established subcutaneous tumor and metastatic models in mice and treated them with the MDM2 inhibitor Nutlin‐3a. As anticipated, Nutlin‐3a significantly suppressed the proliferation of CRC (Figure S7A—C, Supporting Information). Western blotting analysis further demonstrated that Nutlin‐3a upregulated LLGL2 expression while inhibiting the activation of PI3K and Akt (Figure S7D, Supporting Information). Additionally, Nutlin‐3a effectively attenuated the metastasis of CRC cells to the liver (Figure S7E—G, Supporting Information) and lungs (Figure S7H—J, Supporting Information). These results suggest that MDM2 inhibition‐mediated upregulation of LLGL2 represents a potential therapeutic strategy to impede CRC progression.

## 3 Discussion

The function of RBP‐mediated networks in controlling gene expression during the onset and progression of cancer has attracted the interest of researchers.^[^ ^34^ ^]^ RBPs participate in several post‐transcriptional regulatory activities, including translation, RNA splicing, localization, destruction, and modification. The mechanisms by which RBPs regulate the cancer transcriptome are unknown, although over 1500 RBPs have been identified in the human genome.^[^ ^35^, ^36^, ^37^ ^]^ In this study, we also verified that patients with CRC have lower levels of LLGL2 expression, and this decreased expression is linked to the growth and spread of CRC. According to our RNA‐seq and RIP‐seq investigations, LLGL2 function as an RBP that binds to *THBS3* mRNA and recruits CNOT1 to degrade it. This prevents the activation of the PI3K‐Akt pathway, which in turn prevents CRC cells from proliferation and metastasis. Additionally, MDM2, an E3 ubiquitin ligase upstream of LLGL2, can decrease LLGL2 protein expression through ubiquitination modification, thereby promoting the activation of downstream signaling pathways and facilitating CRC progression.

The expression pattern of LLGL2 varies in different cancers. Although LLGL2 expression is reduced in CRC, relevant clinical evidence is still lacking and its specific mechanisms remain unclear.^[^ ^16^ ^]^ Clinical tissue samples and public database data were used to corroborate the decreased LLGL2 expression in CRC. An AOM/DSS‐induced CRC mouse model was used to validate these findings. LLGL2 has been demonstrated to actively facilitate ER^+^ breast cancer.^[^ ^15^ ^]^ However our experimental results indicate that the loss of LLGL2 expression in CRC cells promotes the proliferation, migration, and EMT phenotype. Conversely, LLGL2 overexpression suppressed these phenotypes in CRC cells. This finding was corroborated in multiple animal models. Notably, in the mouse models of metastasis that we constructed, LLGL2 knockdown promoted the formation of distant metastases, whereas overexpression of LLGL2 exhibited a suppressive effect on CRC metastasis.

LLGL2 has been identified as an RBP that interacts with the lncRNA MAYA, via its first three WD40 domains.^[^ ^21^ ^]^ We investigate the role of LLGL2 as an RBP in controlling the expression of downstream RNAs, and their association with the onset and spread of CRC using RNA‐seq and RIP‐seq. Our RIP‐seq results indicated that LLGL2 can bind to multiple RANs, which may underlie its functional role. In conjunction with the RNA‐seq data, we identified *THBS3* as a potential target mRNA that interacts with and is regulated by LLGL2. The TSP family plays a major role in the development and spread of several malignancies.^[^ ^23^, ^24^, ^25^ ^]^ Notably, THBS3 is an extracellular matrix glycoprotein that facilitates cell‐cell interactions.^[^ ^22^ ^]^ THBS1 was identified first and can be readily purified from human platelets, therefore, its role in cancer progression has become more widely understood than that of other TSPs. Previous research has assumed that THBS1, similar to fibronectin, is an extracellular matrix molecule that promotes the migration and adherence of different cell types, including cancer cell lines.^[^ ^38^, ^39^ ^]^ THBS2, functions as a tumor suppressor and is capable of suppressing the proliferation of endothelial cells, triggering apoptosis, and impeding the advancement of the cell cycle.^[^ ^40^ ^]^ Moreover, upon the binding of THBS2 to CD36, a series of events are triggered, including the activation of caspase‐3 and the subsequent reduction in mitochondrial membrane potential. These processes ultimately culminate in the destruction of the epithelial cells.^[^ ^41^ ^]^ Currently, there are few reports on THBS3 expression in tumors, leaving its expression pattern and role in cancer progression unclear. We used RIP‐seq to demonstrate that *THBS3* mRNA is a potential target for binding to the LLGL2 protein, a finding that was confirmed through RIP‐qPCR. Additionally, RNA‐seq results indicated that reduced LLGL2 expression upregulated THBS3 expression, promoting proliferation and metastasis‐related phenotypes in CRC cells. Notably, patients with high THBS3 expression exhibited lower survival rates. GSEA and KEGG analyses suggested that the dysregulated THBS3 expression may activate the PI3K‐Akt pathway, a critical regulator of normal cellular physiological functions,^[^ ^42^ ^]^ thereby facilitating malignant behavior in CRC cells.

Most mRNAs are degraded by shortening of the poly (A) tail through deadacylation.^[^ ^43^, ^44^, ^45^ ^]^ When the Poly (A) tail is removed, the 5ʹ‐cap structure opens up, which causes the mRNA to be degraded by 5ʹ‐3ʹ exonuclease. One of the main necrotic enzymes in mammals, the CCR4‐NOT complex, shortens the mRNA poly(A) tail.^[^ ^46^, ^47^ ^]^ There are a minimum of eight subunits in this complex: CNOT1–3, CNOT6 or CNOT6L, CNOT7 or CNOT8, and CNOT9–11.^[^ ^48^, ^49^ ^]^ CNOT1 functions as a scaffold for RBPs, drawing the complex towards the 3ʹ‐UTR of target mRNAs and subsequently leading to their degradation.^[^ ^30^, ^31^ ^]^ Our mass spectrometry results indicate that LLGL2 interacts with CNOT1, suggesting that this interaction may be the primary mechanism through which LLGL2 facilitates the degradation of THBS3. CNOT1 overexpression counteracted knockdown‐induced increase in THBS3 protein levels brought on by LLGL2 knockdown. In summary, our experimental findings confirm that LLGL2 promotes *THBS3* mRNA degradation by recruiting CNOT1.

The process of ubiquitination is highly selective and involves tagging of substrate proteins and ubiquitin via an ATP‐dependent cascade.^[^ ^50^ ^]^ Furthermore, the ubiquitin‐proteasome system, which controls 80–90% of cellular protein degradation and 10%–20% of autophagy, is formed by ubiquitin and the breakdown by the proteasome.^[^ ^51^ ^]^ Multiple processes are involved in the ubiquitination modification, mediated by ubiquitin‐ligases (E3), ubiquitin‐activating enzymes (E1), and ubiquitin‐conjugating enzymes (E2).^[^ ^52^ ^]^ MDM2 plays a crucial roles in modulating the stability and ubiquitination of various proteins. Initially, it was believed that MDM2 contributed to the ubiquitination of p53 and its subsequent proteasomal destruction.^[^ ^53^ ^]^ With the increasing morbidity and mortality rates of CRC, targeting the interaction between MDM2 and P53 has emerged as a promising research direction.^[^ ^54^, ^55^ ^]^ However, MDM2 interacts with and modifies many other proteins. TCGA database showed a negligible difference in LLGL2 expression between CRC and normal colon tissues. However, data from the CPTAC database revealed that LLGL2 protein levels are significantly lower in CRC tissues. Therefore, we speculated that post‐translational modifications may influence LLGL2 protein expression. Database prediction and experimental confirmation revealed that MDM2 mainly mediates LLGL2 protein degradation. The expression of MDM2 leads to a reduction in LLGL2 protein levels, which explains the observed decrease in LLGL2 expression in CRC at the protein level. This finding complements those of previous studies that focused solely on the transcriptional levels. Additionally, we identified K51 of LLGL2 as the main site of ubiquitination. However, our mutation verification was limited to a few predicted lysine sites, which may have introduced bias and omissions into our data. Nevertheless, building on the work of prior researchers, we have enriched and expanded our understanding of the expression and regulatory patterns of LLGL2 in CRC. Beyond its canonical role in stabilizing p53, our study reveals that Nutlin‐3a exerts antitumor effects in CRC by suppressing MDM2‐mediated ubiquitination of LLGL2, thereby elevating LLGL2 protein levels. In vivo experiments further demonstrate that Nutlin‐3a‐mediated LLGL2 upregulation effectively inhibits CRC proliferation and metastasis. These findings uncover a novel p53‐independent mechanism of Nutlin‐3a, wherein targeting MDM2‐LLGL2 ubiquitination axis represents a promising therapeutic strategy to impede CRC progression. Our work not only expands the understanding of MDM2 inhibitors’ off‐target effects but also provides a compelling rationale for developing next‐generation MDM2 antagonists with dual mechanisms of action.

Plant alkaloids possess diverse structures and biological activities, and are important in both modern and traditional medicine.^[^ ^56^ ^]^ Through molecular docking screening of the major anti‐tumor alkaloids from *Peganum harmala* L., we identified HL as a potential LLGL2‐binding compound. Experimental validation demonstrated that HL could upregulate LLGL2 expression, although its therapeutic efficacy was slightly inferior to that of the MDM2 inhibitor, Nutlin‐3a. These findings suggest that pharmacological intervention targeting the MDM2‐LLGL2 axis, either through MDM2 inhibition or direct LLGL2 activation, represents a promising therapeutic strategy for CRC. The tumor‐suppressive effects mediated by LLGL2 upregulation highlight its potential as a novel molecular target for CRC treatment. Further investigations into the precise mechanisms underlying LLGL2‐mediated tumor suppression may provide additional insights into the developing of targeted therapies against CRC.

## 4 Conclusion

In this study, we discovered that LLGL2 suppresses tumor growth in CRC. We examined the inhibitory effects of normal LLGL2 expression on CRC metastasis, and its ability to suppress CRC progression. RNA‐seq analysis showed that LLGL2 mainly inhibits CRC by repressing PI3K‐Akt activation. Further research using RIP‐seq and shotgun mass spectrometry indicated that LLGL2 inhibits aberrant activation of the PI3K‐Akt pathway by interacting with CNOT1, which stabilizes *THBS3* mRNA. Additionally, we found that LLGL2 was negatively regulated by an E3 ubiquitin ligase. In CRC, MDM2 primarily induces the low LLGL2 expression by promoting the K48‐linked ubiquitination of K51 on LLGL2, thereby facilitating CRC progression. These findings may offer potential therapeutic targets for CRC treatment, as well as strategies for managing CRC progression and metastasis.

## 5 Experimental Section

### Cell Lines and Cell Culture

LOVO, SW620, SW480, HT29, CT26 and RKO cells were obtained from the American Type Culture Collection (ATCC, Manassas, VA, USA). The supplier of HEK293T and HCT116 cells were purchased from Pricella (Wuhan, China). The cells were incubated with 10% fetal bovine serum (FBS; Gibco, Thermo Fisher Scientific, Waltham, MA, USA) and 1% penicillin/streptomycin (Gibco) into RPMI‐1640 and DMEM medium (Pricella, Wuhan, China). The cells were maintain in a humidified incubator set at 37 °C and with 5% CO_2_. All cell lines were examined for *Mycoplasma* contamination, and none were found to be infected.

### CCK8 Assay

Cells (6000) in the logarithmic growth phase were seeded into 96‐well plates. After treating the cells under various conditions, 10 µL of CCK8 (Beyotime, China) solution (in 100 µL of medium) was added per well. The plates were then incubated at 37 °C with 5% CO₂ for 2 h. The absorbance at 450 nm was measured every 24 h for 3 days.

### Colony Formation Assay

A total of 800 cells were seeded per well in six‐well plates. After 24 h of cell attachment, the corresponding treatments were applied. New medium was then added and the plates were incubated for 10–14 days, and the medium was refreshed every 2 days. Upon completion, the cells were fixed with 4% paraformaldehyde for 30 min and the colonies were stained with 0.1% crystal violet. After washing with PBS to remove the excess dye, the stained plates were air‐dried. Finally, pictures of each colony were captured and cell numbers were counted using ImageJ software.

### IF Assay

The CRC cells were seeded in 24‐well plates and incubated overnight. The cells were then washed three times with pre‐chilled PBS, and the medium was discarded. After a 30‐min fixation with 4% paraformaldehyde, three additional washes with PBS were performed. Next, 0.5% Triton X‐100 (Solarbio, Beijing, China) was used to permeabilize the cells, which were then washed three times. The cells were blocked with 10% goat serum for 1 h at room temperature (RT). Antibodies were added and incubated at 4 °C overnight. Subsequently, the cells were treated with either Dylight 488 or Dylight 594 labeled secondary antibodies (Abbkine, Wuhan, China) for 1 h at RT. Finally, nuclei were stained with DAPI (Solarbio) and images were capture using a confocal microscope.

### Clinical CRC Samples Information

Clinical samples from patients with CRC were obtained from the Tianjin Union Medical Center. This study was approved by the Ethics Committee of Tianjin Union Medical Center (2021‐B37). Written informed consent was obtained from all the patients.

### Western Blotting

After disrupting the cells or tissues with RIPA lysate, the protein concentration was accurately quantified. The protein‐loading buffer was then added. Proteins were separated using 8%–15% SDS‐PAGE, and transferred onto a PVDF membrane (Merck Millipore, Burlington, MA, USA). After blocking the PVDF membrane with 5% skim milk powder for 1 h, it was incubated overnight at 4 °C with specific primary antibodies. Subsequently, after a 1‐hour incubation at RT with secondary antibodies, proteins were detected using ECL reagents (ABclonal, Wuhan, China).

### mRNA Stability Assays

To study the effect of LLGL2 on *THBS3* mRNA stability, CRC cells were treated with 0.25 mg mL^−1^ actinomycin D (Selleck, Shanghai, China). Cells were then collected at pre‐set times after treatment, and RT‐qPCR was used to measure *THBS3* mRNA levels.

### RT‐qPCR

Total RNA were extracted from cells and tissues using TRIzol Reagent (ABclonal). The concentrations were then measured., The RNA was then reverse‐ transcribed into cDNA using the PrimeScript RT reagent kit (ABclonal), which served as the amplification template.

### Co‐IP Assay

In the exogenous Co‐IP experiments, CRC or HEK293T cells were pre‐transfected with the designated plasmids. For the endogenous immunoprecipitation (IP) assay, cells cells cultured on 10‐cm plates were lysed with IP buffer containing 50 mM Tris‐HCl (pH 8.0), 150 mM NaCl, 10 mM KCl, 0.5% NP‐40, 10% glycerol, 1 mM EDTA, and protease inhibitors (MCE, NJ, USA). For exogenous Co‐IP, lysed cells were treated with anti‐Flag or anti‐Myc (Proteintech, Wuhan, China), while for endogenous IP, anti‐LLGL2 (Santa Cruz, CA, USA) or anti‐MDM2 was applied. Then, Protein A/G magnetic beads (MCE) were placed on a roller and incubated at 4 °C overnight. The samples were washed six times with PBST. Western blotting was performed on cell lysates and immunoprecipitates to analyze protein interactions.

### RIP‐seq

RIP experiments were conducted using a MagnaRIP RNA Binding Protein Immunoprecipitation Kit (Millipore) instructions. SW620 cells were lysed, with 10% of the lysate set as “input”. Subsequently, 80% of the lysate was immunoprecipitated using an anti‐LLGL2 antibody. Rabbit IgG (CST, Danvers, MA, USA) was added to the remaining 10% as a negative control, labeled “IgG”. RNA from “input” and “IP” was extracted using TRIzol reagent (ABclonal). RNA quality was checked using an Agilent Bioanalyzer2100 system. RNA libraries were prepared using a Truq Stranded RNA Sample Preparation Kit (Illumina). High‐throughput sequencing was performed by SeqHealth (Wuhan, China). Clean reads were normalized for the RPKM calculation.

### RNA‐seq

RNA from SW620‐shNC and SW620‐shLLGL2 cells (three replicates per group) was extracted and sent to SeqHealth for sequencing and analysis. RNA integrity was verified using 1.5% agarose gel electrophoresis and quantified using a QubitTM RNA Broad Range Assay kit (Life Technologies, Q10210). PCR products (200–500 bps) were enriched, quantified, and sequenced on a DNBSEQ‐T7 sequencer.

### HE and IHC Analysis

For Hematoxylin and eosin (HE), tissues were fixed in 4% paraformaldehyde. The tissues were then dehydrated, paraffin embedded, and sectioned, prior to staining with an H&E kit (Sorlabio). For Immunohistochemistry (IHC), samples were treated with anti‐LLGL2 (1:200) and anti‐Ki67 (1:200) antibodies following the IHC kit (Sorlabio) instructions. Subsequently, The cells were then imaged under a microscope.

### Mouse Colon Infected with AAV

Mice were fasted overnight and subsequently anesthetized using isoflurane. Following anesthesia, a rectal injection was administered, delivering 1×10^11^ physical particles of AAV (Genechem, Shanghai, China) suspended in 300 µL of PBS into the intestinal tract.

### Animal Models

Male BALB/c mice (5 weeks old) were obtained from Beijing Vital River Laboratory Animal Technology Co. Ltd. and randomly grouped. The experiments strictly adhered to the Guidelines for the Care and Use of Laboratory Animals (2006) of the Chinese Ministry of Science and Technology and were approved by the relevant committees at Tianjin University of Traditional Chinese Medicine (approval numbers: TCM‐LAEC2021242 and TCM‐LAEC2022170).

For the AOM/DSS‐induced CRC mouse model, AOM (Sigma‐Aldrich, USA) was injected into the mice one week before the experiment. The mice then received one week of drinking water with 2.5% DSS (Yeasen, Shanghai, China), followed by two weeks of regular drinking water. During the three repetitions of this cycle, the body weight and fecal occult blood of the mice were measured every two days. Following the completion of the three cycles, the mice were allowed to sleep and the tissues from their colons were collected for further study.

For mice homograft tumor model, CT26 cells (5 × 10^5^) that had undergone differential treatment were isolated and resuspended in 200 µL of PBS for the subcutaneous homograft tumor model. Subsequently, the animals received subcutaneous injections of the cells. To track tumor size and determine tumor volume (V), calipers were used to measure tumor length (L) and width (W) every two days. The formula for the computation was V = 1/2 × L × W^2^. Mice were euthanized approximately 4–5 weeks after injection, and tumor weights were recorded.

For the mice CRC‐metastasis model, stabilized CT26 cells infected with various lentiviruses were gathered and resuspended in PBS at a concentration of 5 × 10^5^ cells per 100 µL of PBS for the CRC metastatic model. A syringe was used to inject the cell solution into the tail vein of the mice in the CRC model with lung metastases. Lung metastatic nodules were counted and the weight of the mouse lung tissue was recorded. First, the mouse was anesthetized and the spleen was exposed. Then, a syringe was used to inject 100 µL of cell suspension into the mouse spleen. Finally, the spleen was repositioned and the mouse skin was stitched to create the CRC liver metastasis model. Approximately four weeks later, the mice were euthanized after their body weights were measured. Subsequently, the livers were removed, and weighed. In addition, metastatic liver nodules were statistically recorded.

### Statistical Analysis

Statistical analyses were performed using GraphPad Prism 8.0. software. The correlation between MDM2 and LLGL2 was performed by Pearson correlation analysis. For the comparisons between the two groups, two‐tailed Student's *t*‐tests were used. ANOVA was used for multiple comparisons among more than two groups. N ≥ 3 in each group. The data were shown as mean ± SD, and P < 0.05 was considered statistically significant.

## Conflict of Interest

The authors declare no conflict of interest.

## Author Contributions

The study was designed by J.H. and H.Y. The implementation was carried out by J.H., T.Z., and H.L. Z.L., S.Y., and Y.L. assisted with the acquisition of tissue samples. C.Z., and Y.Q. devised the experimental methodologies and conducted The manuscript was initially drafted by J.H. and H.Y.; J.H. oversaw the illustrations. Y.Q., and H.Y. reviewed the study and provided financial resources. All the authors participated in the review and gave their approval to the final version of the manuscript.

## Supporting information



Supporting Information

## Data Availability

The data that support the findings of this study are available from the corresponding author upon reasonable request.
