# Supplementary material for: Ubiquitination‐Dependent LLGL2 Degradation Drives Colorectal Cancer Progression via THBS3 mRNA Stabilization
Source: Adv Sci (Weinh). 2025 Jul 6;12(39):e01656. doi: 10.1002/advs.202501656 (PMC12533200; doi:10.1002/advs.202501656)
Supplement: Supplementary file 1 — Supporting Information [file ADVS-12-e01656-s001.docx]

**Supporting Information**

**Ubiquitination-Dependent LLGL2 Degradation Drives Colorectal Cancer Progression *via THBS3* mRNA Stabilization**

**Authors:** *Jiayan Huang^1,2^, Tiantian Zhang^1,2^, Huimin Li^1,2^, Zidan Li^1,2^, Shuangshuang Yin^1,2^, Yiman Liu^1,2^, Chunze Zhang^3^, Yuling Qiu^4^*, Haiyang Yu^1,2^*.*

**Affiliations:** ^1^ State Key Laboratory of Chinese Medicine Modernization, Tianjin University of Traditional Chinese Medicine, Tianjin, 301617, China. ^2^ Haihe Laboratory of Modern Chinese Medicine, Tianjin, 301617, China. ^3^ Department of Colorectal Surgery, Tianjin Union Medical Center, Nankai University, Tianjin, 300122, China. ^4^ School of Pharmacy, Tianjin Medical University, Tianjin, 300070, China.

***Correspondence:** Haiyang Yu, State Key Laboratory of Chinese Medicine Modernization, Tianjin University of Traditional Chinese Medicine, Tianjin, 301617, China; Haihe Laboratory of Modern Chinese Medicine, Tianjin, 301617, China. Tel: +86 22 5959 6163; Fax: +86 22 5959 6163; E-mail: hyyu@tjutcm.edu.cn.

Yuling Qiu, School of Pharmacy, Tianjin Medical University, Tianjin, 300070, China. Tel: +86 22 8333 6658; Fax: +86 22 8333 6560; E-mail: [qiuyuling@tmu.edu.cn](mailto:qiuyuling@tmu.edu.cn).


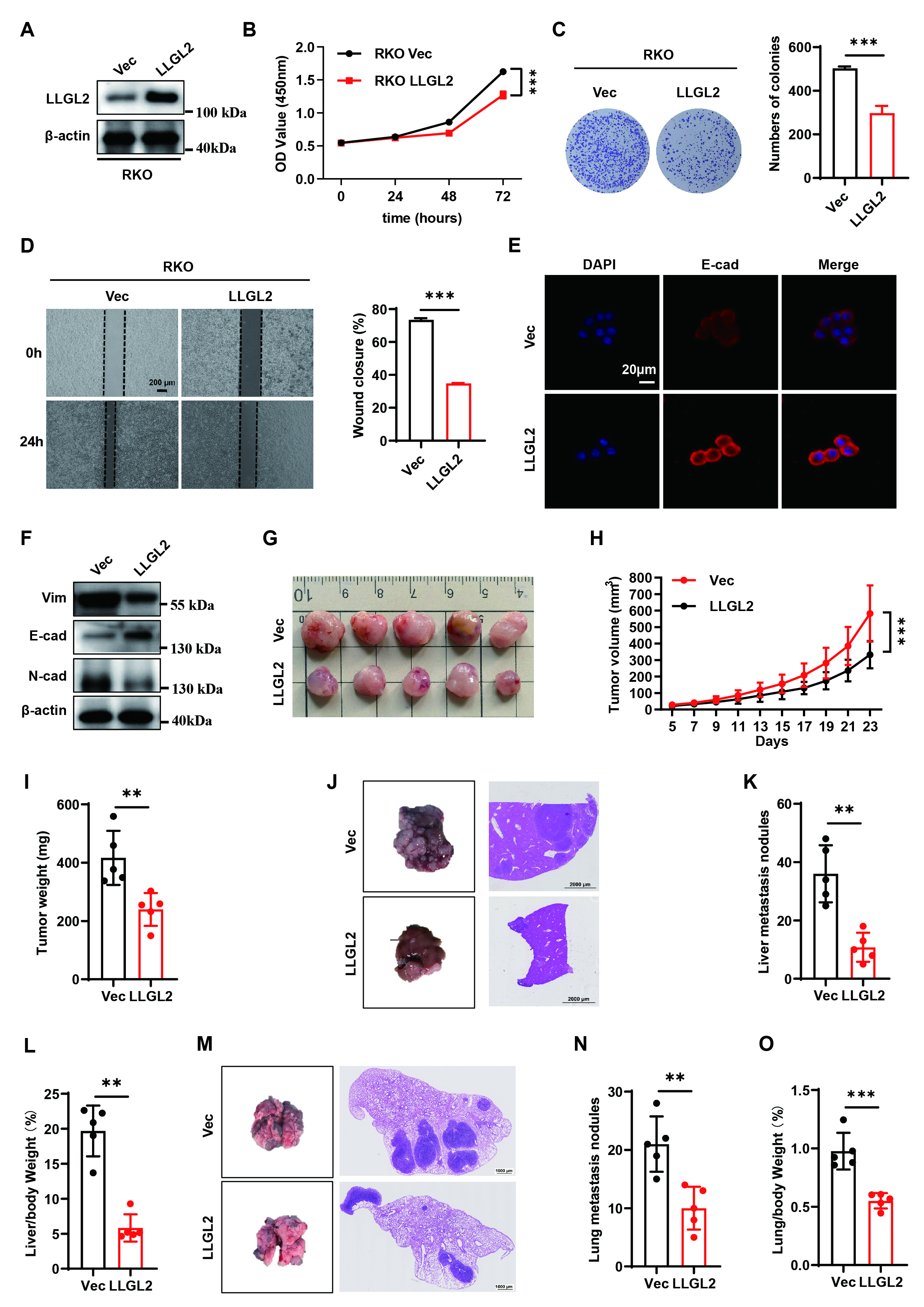


**Figure S1** The overexpression of LLGL2 inhibits the proliferation and migration capabilities of CRC cells both *in vivo* and *in vitro*. **A** Western blotting analysis of LLGL2 overexpression efficiency in RKO cells. **B** CCK8 assay of RKO cells overexpressing LLGL2. **C** Clone formation assay of RKO cells overexpressing LLGL2. **D** Wound healing assay of RKO cells overexpressing LLGL2. **E** IF staining of E-cad in RKO cells overexpressing LLGL2. Scale bar, 20 µm. **F** Western blotting analysis of Vim, E-cad, and N-cad protein expression in RKO cells overexpressing LLGL2. **G** Image of tumors in Vec and LLGL2 group. **H** Volume variations during tumor growth in Vec and LLGL2 groups (n = 5 per group). **I** Statistics of subcutaneous tumor weight in Vec and LLGL2 groups (n = 5 per group). **J** Image of liver metastasis tumors are provided in Vec and LLGL2 groups, along with HE staining. Scale bar, 2000 µm. **K** Statistics regarding the quantity of liver metastasis tumor nodules in Vec and LLGL2 groups (n = 5 per group). **L** The liver-to-body weight ratio of mice in Vec and LLGL2 groups (n = 5 per group). **M** Image of lung metastasis tumors are provided in Vec and LLGL2 groups, along with HE staining. Scale bar, 1000 µm. **N** Statistics regarding the quantity of lung metastasis tumor nodules in Vec and LLGL2 groups (n = 5 per group). **O** The lung-to-body weight ratio of mice in Vec and LLGL2 groups (n = 5 per group). Data are presented as mean ± SD. *P*-values are determined by a two-tailed Student’s *t*-test or two-way ANOVA. ***P* < 0.01, ****P* < 0.001.


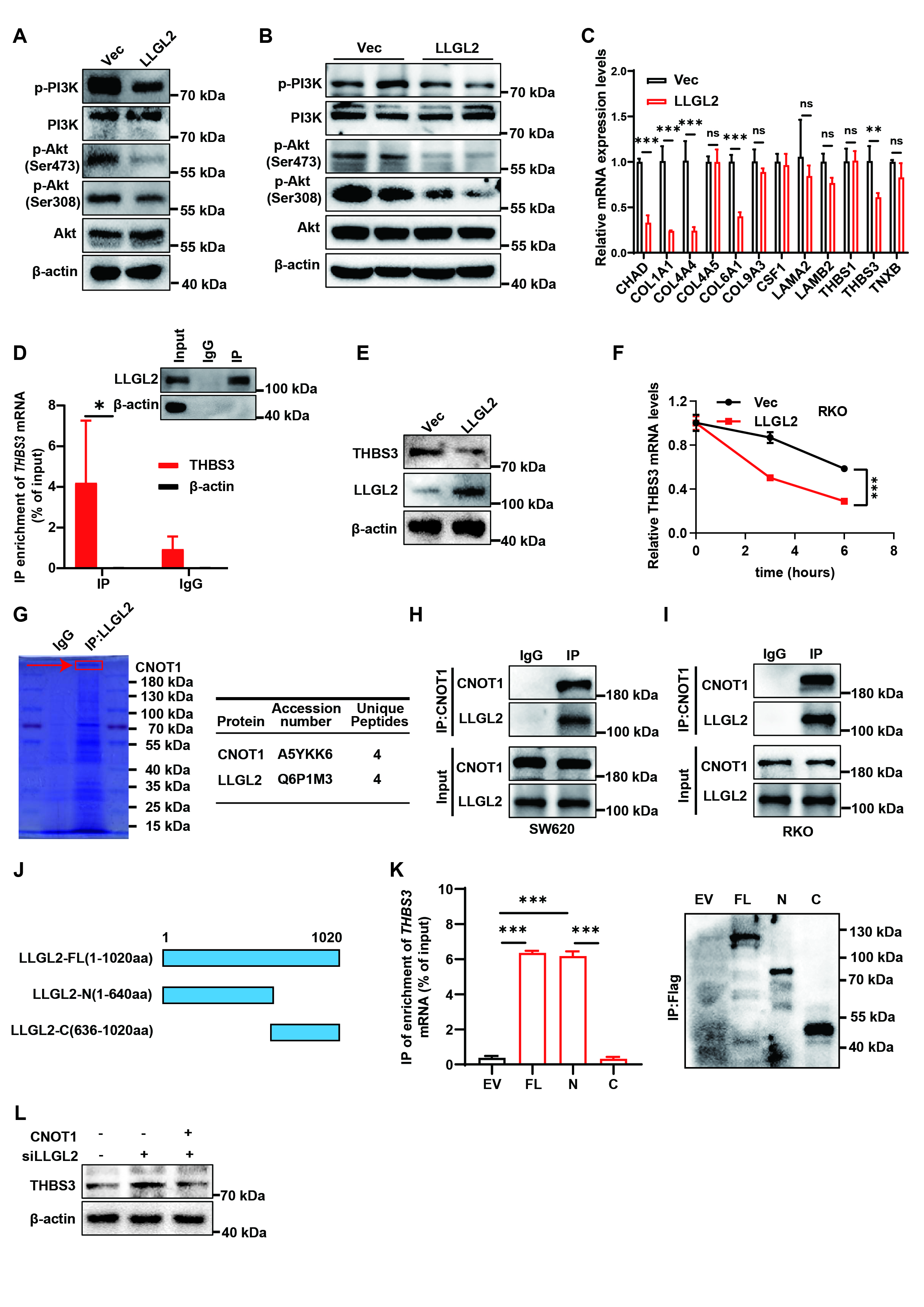


**Figure S2** LLGL2 binds to *THBS3* mRNA and affects its stability. **A** Western blotting analysis of p-PI3K and p-Akt expression in RKO cells overexpressing LLGL2. **B** The p-PI3K and p-Akt in subcutaneous tumor tissues of Vec and LLGL2 mice were analyzed. **C** RT-qPCR analysis of 12 PI3K-Akt pathway genes in RKO cells overexpressing LLGL2. **D** RIP-qPCR analysis of endogenous LLGL2 and *THBS3* mRNA binding in RKO cells. **E** Western blotting analysis of THBS3 expression in RKO cells with LLGL2 overexpression. **F** RT-qPCR analysis of *THBS3* mRNA half-life in RKO cells overexpressing LLGL2. **G** SDS-PAGE and coomassie brilliant blue staining of LLGL2 immunoprecipitated proteins from LLGL2-overexpressing SW620 cells, with arrows marking the proteins of interest. **H** LLGL2-CNOT1 interaction in SW620 cells. Co-IP assays using anti-CNOT1 (IP) versus IgG control. **I** LLGL2-CNOT1 interaction in RKO cells. Co-IP assays using anti-CNOT1 (IP) versus IgG control. **J** Schematic representation of LLGL2 domain deletion mutants. **K** RIP-qPCR analysis of the binding capacity between LLGL2 mutants and *THBS3* mRNA. **L** THBS3 expression by western blotting upon LLGL2 knockdown with/without CNOT1 overexpression. Data are presented as mean ± SD. *P*-values are determined by a two-tailed Student’s *t*-test or one/two-way ANOVA. **P* < 0.05, ***P* < 0.01, ****P* < 0.001.


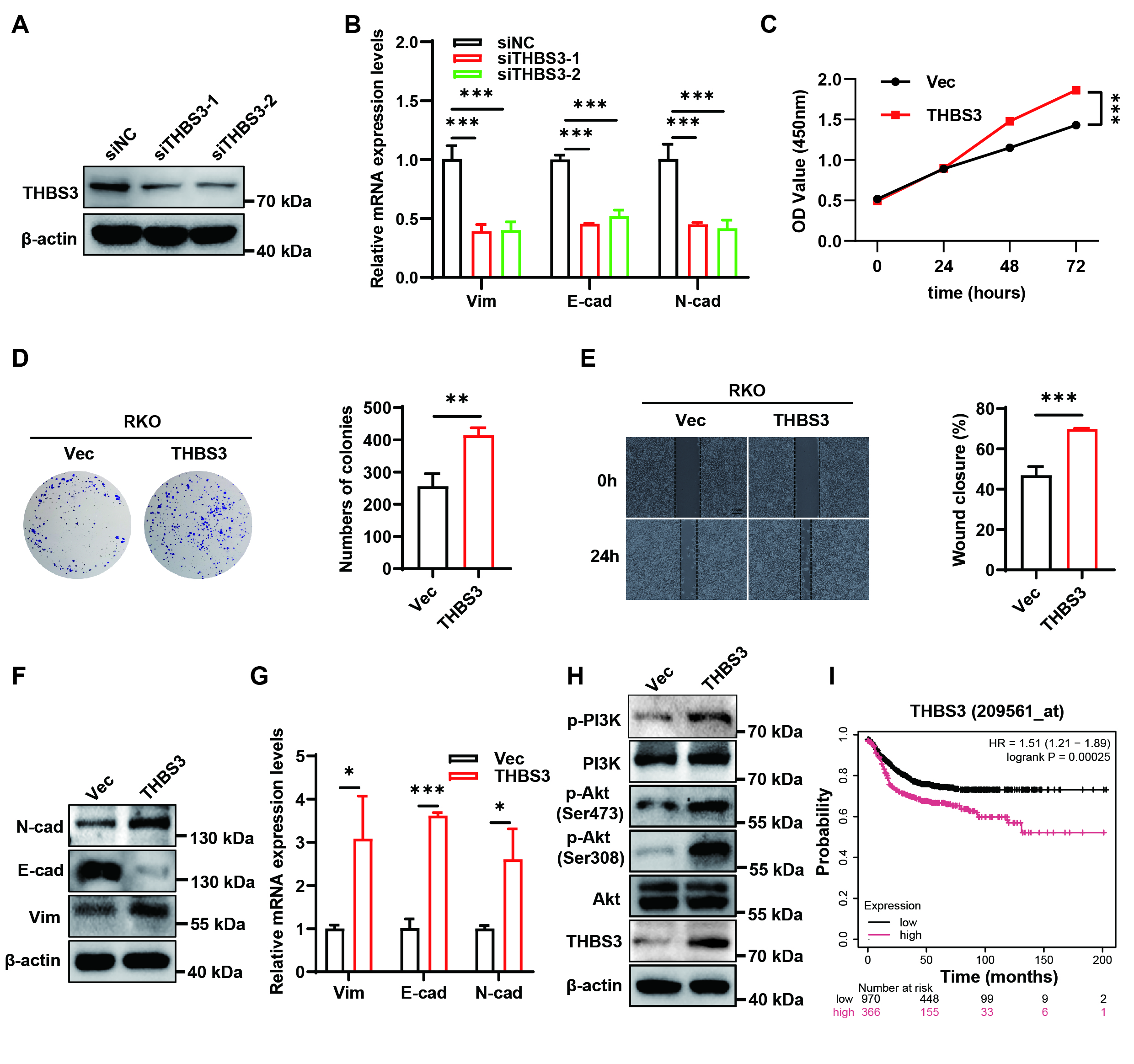


**Figure S3** THBS3 activates PI3K and Akt to promote CRC cell proliferation and migration. **A** Validation of THBS3 knockdown efficiency by western blotting. **B** RT-qPCR analysis of EMT markers (Vim, E-cad, N-cad) after THBS3 knockdown. **C** CCK-8 proliferation assay of THBS3-overexpressing RKO cells. **D** Colony formation assay of THBS3-overexpressing RKO cells. **E** Wound healing assay of THBS3-overexpressing RKO cells. **F** Western blotting analysis of EMT markers (E-cad, Vim, N-cad) in THBS3-overexpressing RKO cells. **G** RT-qPCR analysis of EMT markers (E-cad, Vim, N-cad) in THBS3-overexpressing RKO cells. **H** Western blotting analysis of p-PI3K and p-Akt expression in THBS3-overexpressing RKO cells. **I** Kaplan–Meier analysis of RFS in patients with high vs. low THBS3 expression. Data are presented as mean ± SD. *P*-values are determined by a two-tailed Student’s *t*-test or one/two-way ANOVA. **P* < 0.05, ***P* < 0.01, ****P* < 0.001.


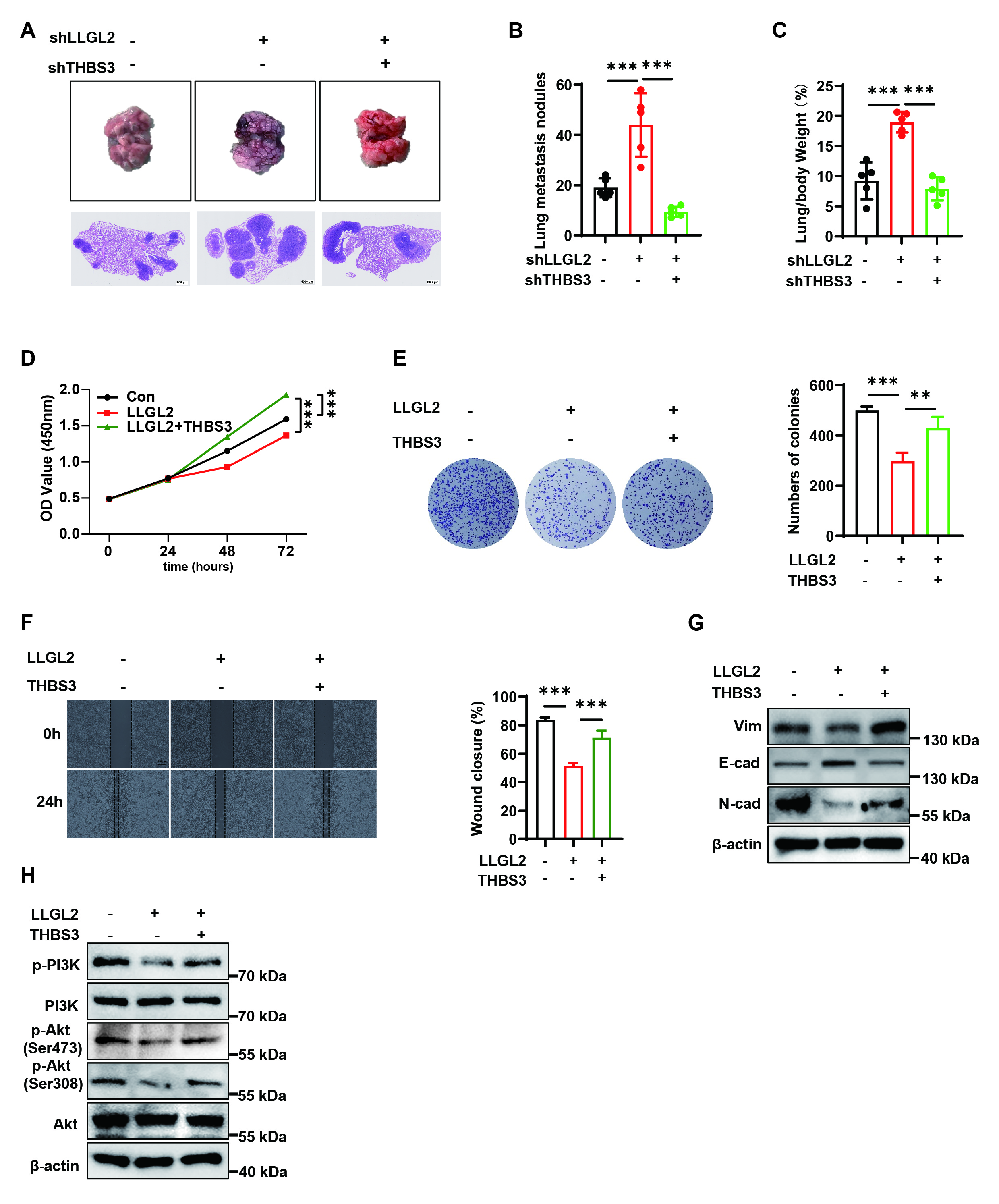


**Figure S4** LLGL2 impacts the progression of CRC through modulating the PI3K-Akt pathway *via* THBS3. **A** Representative images of lung tumors from the control group, LLGL2 knockdown group, and LLGL2/THBS3 double-knockdown group. **B** Quantitative analysis of lung metastasis nodules across experimental groups (n = 5). **C** Statistical analysis of lung-to-body weight ratio among different groups (n = 5). **D** CCK-8 assay of RKO cells with LLGL2 or LLGL2/THBS3 overexpression. **E** Colony formation of RKO cells with LLGL2 or LLGL2/THBS3 overexpression. **F** Wound healing assay of RKO cells with LLGL2 or LLGL2/THBS3 overexpression. **G** Western blotting analysis of EMT-related proteins (E-cad, Vim, N-cad) expression in RKO cells with LLGL2 or LLGL2/THBS3 overexpression. **H** Western blotting analysis of PI3K/Akt activation in RKO cells overexpressing LLGL2 alone or in combination with THBS3. Data are presented as mean ± SD. *P*-values are determined by one-way ANOVA. ***P* < 0.01, ****P* < 0.001.


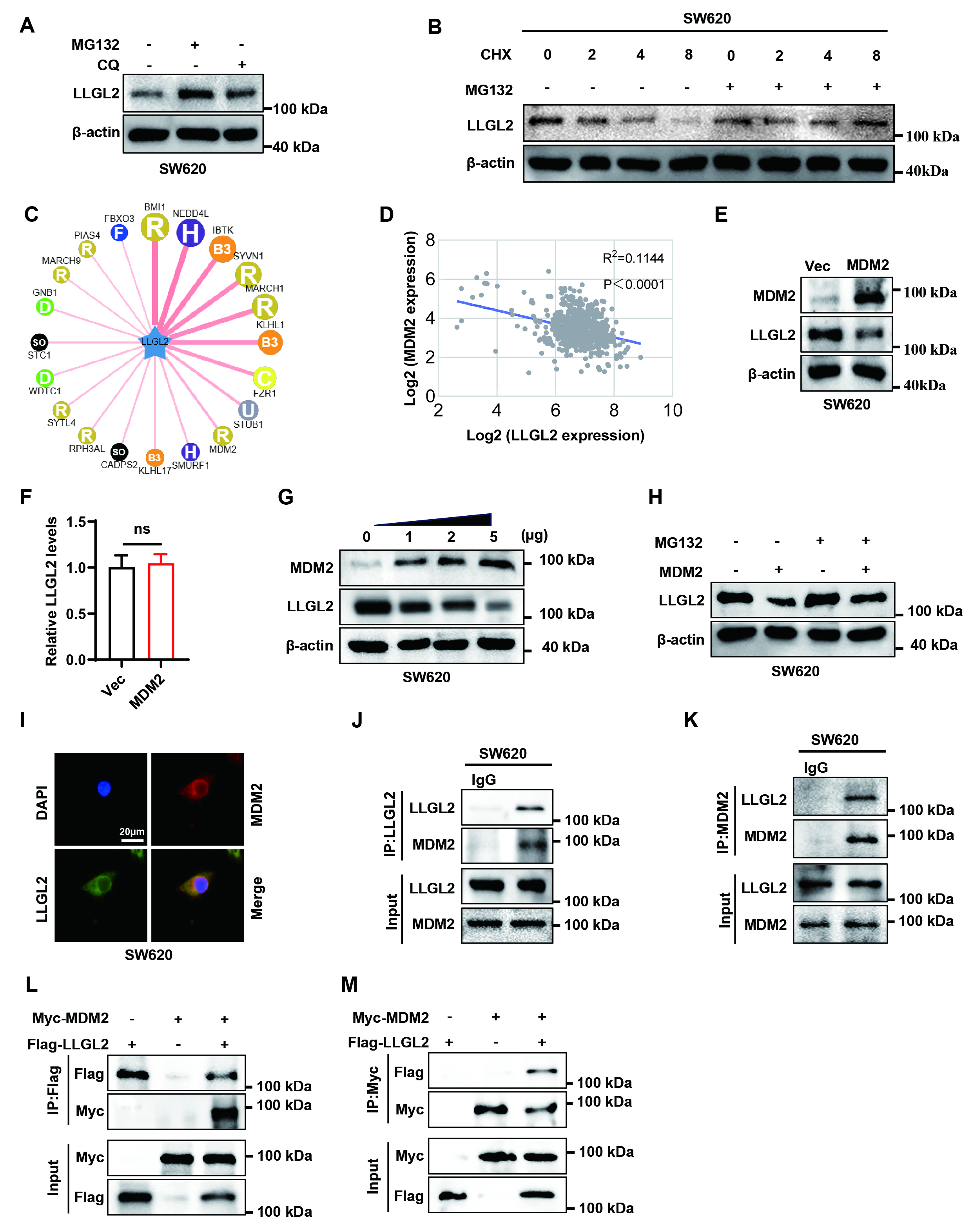


**Figure S5** MDM2 engages in interaction with LLGL2 and modulates its levels. **A** Western blotting analysis of LLGL2 protein in SW620 cells post-treatment with 10 μM MG132 or 20 μM CQ. **B** Western blotting analysis of LLGL2 protein changes in SW620 cells treated with 50 μg mL^-1^ CHX for varying durations, with or without 10 μM MG132. **C** Ubibroswer database was used to predict LLGL2-associated ubiquitinated E3 ligases. **D** Correlation analysis of LLGL2 and MDM2 expression in CRC patients from the TCGA database. **E** Western blotting analysis of LLGL2 protein expression in SW620 cells overexpressing MDM2. **F** RT-qPCR analysis of LLGL2 mRNA levels in SW620 cells overexpressing MDM2. **G** Western blotting analysis of LLGL2 protein levels in SW620 cells following transfection with varying doses of MDM2 plasmid. **H** Western blotting analysis of LLGL2 protein levels in SW620 cells following the overexpression of MDM2 expression, with and without treatment with 10 μM MG132. **I** Colocalization of MDM2 and LLGL2 in SW620 cells by IF. **J,K** Co-IP analysis of endogenous MDM2 and LLGL2 interaction in SW620 cells. **L,M** Flag-LLGL2 and Myc-MDM2 were transfected into SW620 cells, and a Co-IP assay was conducted to examine the interaction between Flag-LLGL2 and Myc-MDM2.


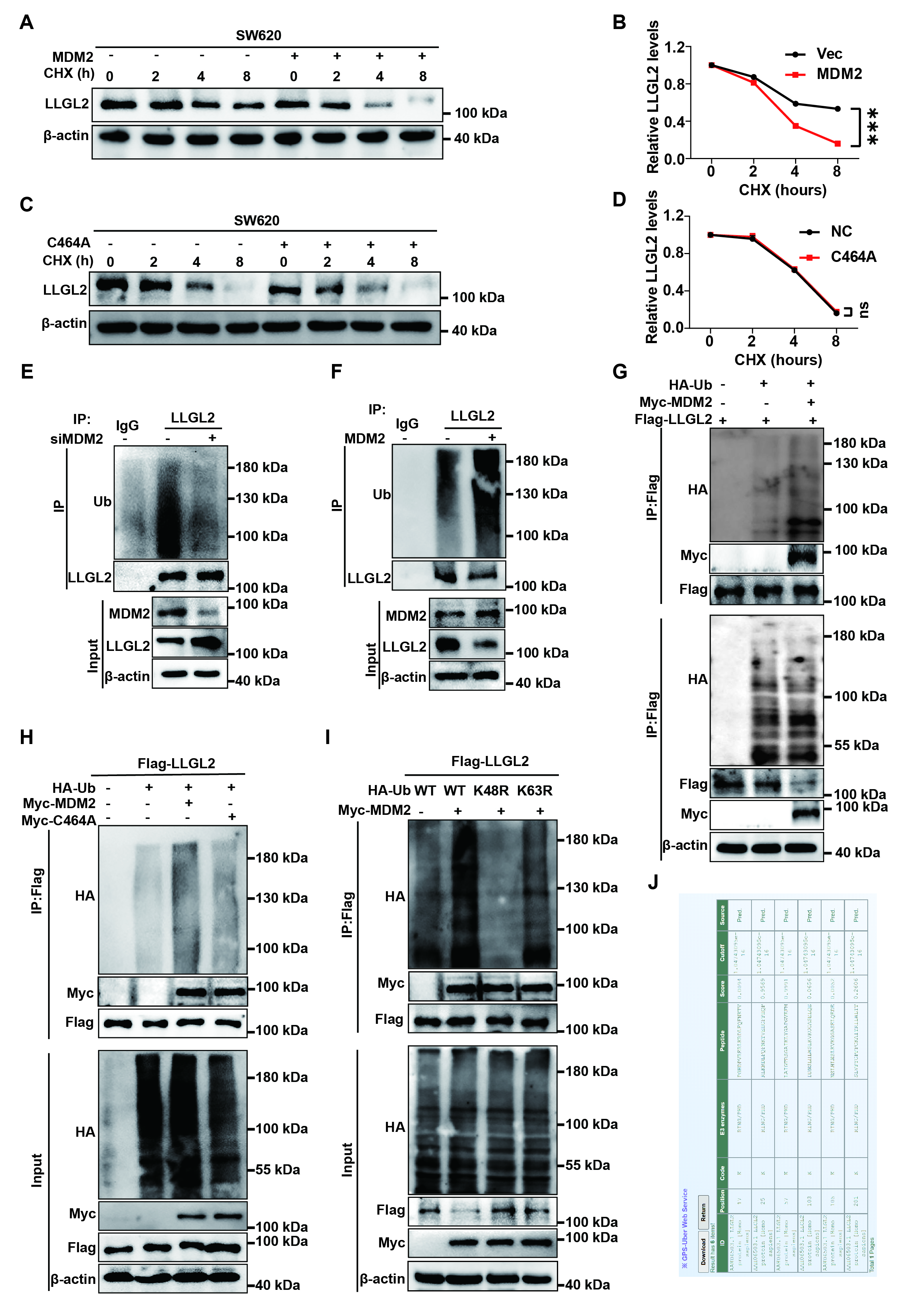


**Figure S6** MDM2 functions as an E3 ubiquitin ligase, facilitating the degradation of the LLGL2 protein. **A** Western blotting analysis of LLGL2 in MDM2-overexpressing SW620 cells treated with CHX for indicated durations. **B** Quantification of LLGL2 protein in control and MDM2 overexpression groups treated with CHX for indicated times. **C** Western blotting analysis of LLGL2 expression in SW620 cells transfected with/without C464A plasmid followed by CHX treatment **D** Quantification of LLGL2 protein in control and C464A overexpression groups treated with CHX for indicated times. **E** Ubiquitination levels of LLGL2 were examined by western blotting in MDM2-knockdown RKO cells. **F** Ubiquitination levels of LLGL2 was assessed by western blotting in MDM2-overexpressing SW620 cells. **G** SW620 cells were transfected with HA-Ub, Myc-MDM2, and Flag-LLGL2 plasmids. A Flag antibody was utilized for the Co-IP experiment, while western blotting analysis was employed to assess the expression levels of HA-Ub. **H** SW620 cells were transfected with HA-Ub, Myc-MDM2, Flag-LLGL2, and Myc-C464A plasmids. A Flag antibody was used for the Co-IP experiment, and western blotting was conducted to analyze the expression levels of HA-Ub. **I** SW620 cells were co-transfected with Myc-MDM2, Flag-LLGL2, HA-Ub and its different mutants. Flag antibody was used for Co-IP experiment, and western blotting experiment was used to analyze the expression level of HA-Ub. **J** Lysine sites for potential ubiquitination of LLGL2 protein predicted by the GPS-Uber database. Data are presented as mean ± SD. *P*-values are determined by two-way ANOVA. ****P* < 0.001, ns (not significant).


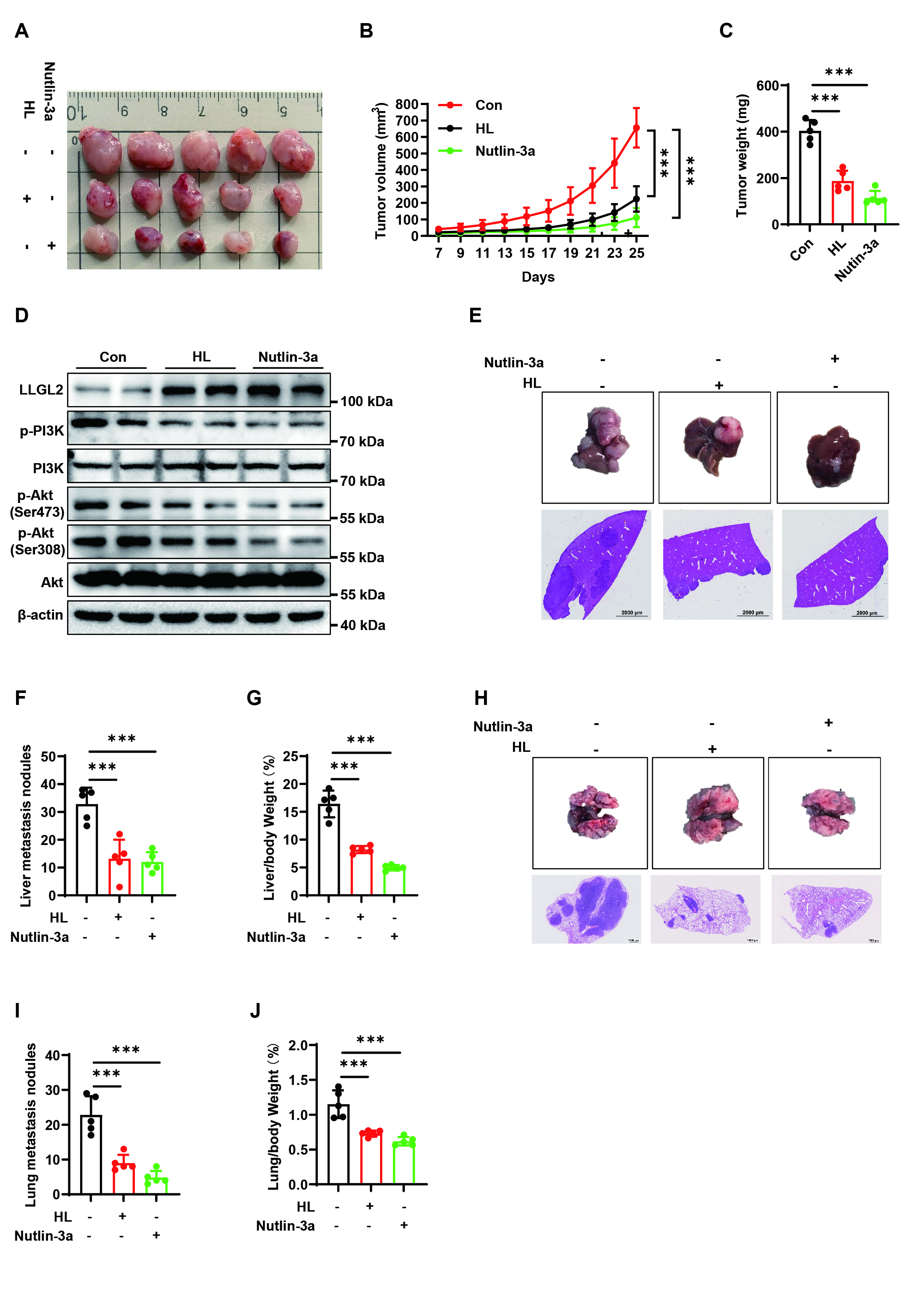


**Figure S7** The natural compound Harmalol (HL) inhibits CRC *via* LLGL2 activation. CT26 cells were injected into the axilla of BALB/C mice, and treated with HL and Nutlin-3a at 50 mg kg^-1^, i.p., every two days. **A** Representative tumor images from different groups. **B** Tumor volume changes in different groups (n = 5 per group). **C** Tumor weight. **D** Western blotting analysis of LLGL2 expression changes and PI3K and Akt activation following HL and Nutlin-3a treatment. **E** Representative images of liver metastatic tumors in mice treated with HL and Nutlin-3a. **F** Statistical analysis of the number of liver metastatic nodules (n = 5 per group). **G** Statistical analysis of the liver-to-body weight ratio. **H** Representative images of lung metastatic tumors in mice treated with HL and Nutlin-3a. **I** Statistical analysis of the number of lung metastatic nodules. **J** Statistical analysis of the lung-to-body weight ratio. Data are presented as mean ± SD. *P*-values are determined by one/two-way ANOVA to compare multiple groups, ****P* < 0.001.
